# DDX3x Regulates NINJ1 Transcription via Histone Lactylation in Sepsis Associated‐Acute Kidney Injury

**DOI:** 10.1002/advs.76979

**Published:** 2026-08-17

**Authors:** Hongyu Liang, Jihong Jiang, Huanxin Yin, Chunyan Liu, Hongjun Huang, Yan Luo, Minmin Zhu, Qiuyun Wang

**Affiliations:** ^1^ Department of Anesthesiology Ruijin Hospital Shanghai Jiao Tong University School of Medicine Shanghai China; ^2^ Department of Anesthesiology Shanghai General Hospital Shanghai Jiao Tong University School of Medicine Shanghai China; ^3^ Shanghai Key Laboratory of Anesthesiology and Brain Functional Modulation School of Medicine Shanghai Fourth People's Hospital Tongji University Shanghai China; ^4^ Department of Anesthesiology Shanghai Pudong Hospital Fudan University Pudong Medical Center Shanghai China

**Keywords:** cell biology, histone H3, membrane protein, programmed cell death

## Abstract

Sepsis associated‐acute kidney injury (SA‐AKI), a severe complication of sepsis, is characterized by impaired tubular injury that can ultimately cause renal failure and patient mortality. Both histone and non‐histone lactylation and cellular PANoptosis have been implicated in the pathogenesis of SA‐AKI. In this study, we investigated how lactylation in histone H3 at lysines 9, 18, and 27 (H3K9/18/27la) modulates PANoptosis in SA‐AKI. Our findings revealed that lactate triggers PANoptosis by promoting H3K9/18/27la through the activation of Ninjurin‐1 (NINJ1) gene transcription in SA‐AKI. Notably, NINJ1, a membrane protein, is also lactylated at residues K111 (K111la) and K114 (K114la) in lipopolysaccharides (LPS)‐induced HK‐2 cells. NINJ1‐K111la mediates plasma membrane localization, thereby promoting cell death. Notably, we identified DEAD (Asp‐Glu‐Ala‐Asp)‐box helicase 3x (DDX3x), a previously unreported delactylase capable of regulating H3K9/18/27la levels, which influences NINJ1 transcription and PANoptosis. Furthermore, we developed Odetiglucan (Ode), a novel agonist targeting DDX3x delactylase function. Application of Ode in cecum ligation and puncture (CLP) mice or LPS‐induced HK‐2 cells significantly reduced H3K9/18/27la levels, inhibited NINJ1 transcription and PANoptosis, and alleviated SA‐AKI progression. In summary, DDX3x, function as a delactylase, regulates PANoptosis by influencing H3K9/18/27la. Thus, enhancing the delactylase activity of DDX3x may represent a potential therapeutic strategy for SA‐AKI.

## Graphical abstract

This study identifies a potential therapeutic approach for sepsis‐associated acute kidney injury (SA‐AKI). We found that during SA‐AKI, reduced expression of DDX3x in renal tubular epithelial cells mediates histone delactylation, which in turn upregulates NINJ1 transcription and triggers tubular cell death. Conversely, Odetiglucan confers protection by inhibiting the histone delactylation activity of DDX3x.

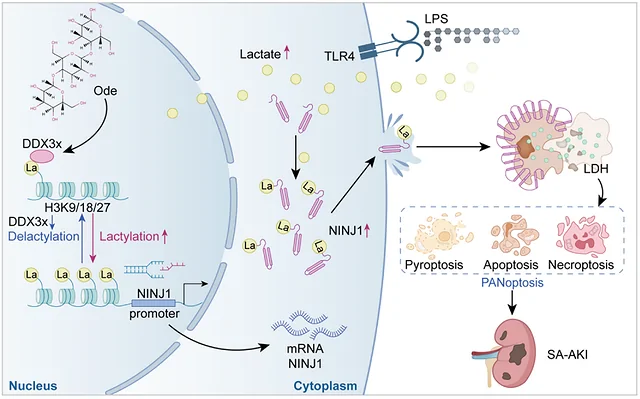

## 1 Introduction

Sepsis is defined as a life‐threatening organ dysfunction caused by a dysregulated host response to infection, with approximately 90% of ICU patients exhibiting varying degrees of sepsis [1]. Acute kidney injury (AKI) frequently complicates critical illness. The diagnostic criteria for AKI primarily rely on various serum markers of renal injury (e.g., an increase in serum creatinine of ≥50% within 7 days) [2]. Many patients concurrently meet the consensus criteria for both sepsis and AKI and are thus considered to have sepsis associated ‐acute kidney injury (SA‐AKI) [3, 4]. Sepsis accounts for up to half of AKI cases, whereas SA‐AKI develops in approximately 60% of patients with sepsis [5]. Mechanistically, SA‐AKI primarily arises from varying degrees of structural or functional alterations in renal tubular cells, and recent researches revealed the role of cell death in SA‐AKI pathogenesis [6]. Current treatments for SA‐AKI rely primarily on anti‐infective or anti‐inflammatory therapies. However, only a limited number of drugs have progressed to clinical trials. Consequently, investigating novel molecular mechanisms underlying SA‐AKI progression and identifying promising drug targets are of paramount importance.

A variety of diseases are significantly influenced by forms of regulated cellular death, most notably necroptosis, apoptosis, and pyroptosis [7]. Recent studies have indicated that renal tubular epithelial cell death is a core driver of the SA‐AKI pathological process [8, 9]. Extracellularly discharged damage‐associated molecular patterns (DAMPs) following cell death act as catalysts for inflammatory cascades, culminating in an intractable “cell death‐inflammatory storm”. Our previous research demonstrated the distinct roles and mechanisms of multiple cell death pathways during sepsis [10]. NINJ1 acts as an executor of cell death and has previously been regarded as a neurotrauma‐induced protein. Recent studies have reported its role in mediating plasma membrane rupture during cell death [11, 12, 13], leading to the release of DAMPs and the subsequent induction of a form of cell death termed PANoptosis. PANoptosis is a novel form of cell death that integrates three programmed cell death (PCD) pathways including necroptosis, apoptosis, and pyroptosis. Recent studies have reported a potential role for NINJ1‐driven PANoptosis in infection [14, 15]. Therefore, elucidating the specific regulatory mechanisms of PANoptosis in SA‐AKI may aid in the development of novel therapeutic strategies.

Varying degrees of metabolic reprogramming occur during SA‐AKI, which is primarily characterized by reduced aerobic phosphorylation and increased glycolysis [16]. Lactate, the end‐product of glycolysis, is also a serum marker of sepsis. In 2019, Zhao et al. first demonstrated that lactate, acting as a substrate, can induce lactylation of lysine residues of proteins through the action of multiple lactyltransferases /delactylases [17]. Lactate‐mediated protein lactylation contributes to organ damage in sepsis [18]; however, the precise mechanism by which it induces ANoptosis in septic renal tubular epithelial cells remains unclear. Furthermore, the lactyltransferases/delactylases that mediate lactylation during SA‐AKI require further identification.

The DEAD (Asp‐Glu‐Ala‐Asp)‐box helicase (DDX) family constitutes the largest helicase family in eukaryotes and participates in virtually every aspect of eukaryotic RNA metabolism [19]. DDX3 is a major family of DDX and is encoded in humans by two genes: *DDX3x* and *DDX3y* [20]. DDX3x is primarily localized on the X chromosome, exhibiting widespread expression across organs and high evolutionary conservation. Functionally, DDX3x regulates RNA metabolism, including transcription to translation [21, 22]. DDX3x dysfunction plays a significant role in various diseases, including cancer and developmental defects [23, 24]. Recent studies highlighted the role of DDX3x in cell death [25]. Our group has previously demonstrated the importance of DDX3x in the innate antiviral immune response [26]. Furthermore, recent bioinformatics studies have indicated a potential regulatory role for DDX3x in PANoptosis in patients with sepsis [27].

Here, we identified DDX3x as a potential delactylase that promotes histone H3 delactylation both in vitro and in vivo. Furthermore, we observed reduced DDX3x expression in SA‐AKI, which enhanced NINJ1 transcription by increasing H3 lysine 9, 18, 27 lactylation (H3K9/18/27la), thereby inducing PANoptosis. Notably, we also identified a DDX3x agonist—Odetiglucan (Ode), and validated that Ode modulates DDX3x delactylases activity in SA‐AKI. This study revealed a novel mechanism of cell death during SA‐AKI and explored a potential therapeutic approach for SA‐AKI that may help reduce or prevent renal injury in patients with sepsis.

## 2 Results

### 2.1 Lactate Levels, Pan‐lysine Lactylation (Pan‐Kla), and H3K9/18/27la Increased in SA‐AKI

We characterized renal pathological changes in a cecal ligation and puncture (CLP) model of SA‐AKI. Hematoxylin‐Eosin (H&E) staining and Masson staining revealed swelling and necrosis in the renal tubules of CLP mice, alongside elevated levels of IL‐1β and TNF‐α, compared to that in Sham group. Serum creatinine (Scr) and blood urea nitrogen (BUN), two renal injury markers, also exhibited varying degrees of increase, indicating the successful induction of SA‐AKI (Figure 1A–C). Prior research indicates that metabolic reprogramming within the kidney during SA‐AKI leads to increased glycolysis [28, 29]. We examined the expression of key glycolytic enzymes and observed varying degrees of elevation in the hexokinase‐1 (HK‐1), phosphofructokinase platelet (PFKP), and lactate dehydrogenase A (LDHA) levels in the CLP group (Figure 1D). This finding was corroborated by the publicly available single‐cell databases (GSE95233), which confirmed other specific upregulation of glycolysis‐related genes (Figure S1A). Lactate, the end product of glycolysis, was found to be elevated in the kidneys of CLP mice through metabolomic analysis (Figure 1E). The direct measurement of renal lactate levels further corroborated the metabolomic findings (Figure 1F). Previous studies have reported the potential role of lactate‐mediated lactylation in sepsis or SA‐AKI [28, 30]. Consistent with these researches, our results also demonstrated upregulated Pan‐lysine lactylation (Pan‐Kla) levels in CLP mice (Figure 1G). Given that histone modifications are key regulators of gene transcription, we performed fluorescence co‐localization of histones H1/H2A/H2B/H3/H4 with Pan‐Kla in CLP mouse kidneys, and the results showed strong co‐localization between H3 and Pan‐Kla (Figure 1H). Western blot and immunohistochemistry (IHC) results also revealed that in CLP mouse kidney tissue, H3K9/18/27la exhibited varying degrees of increase, whereas H3K14/23la showed no significant alteration (Figure 1I,J).

**FIGURE 1:**
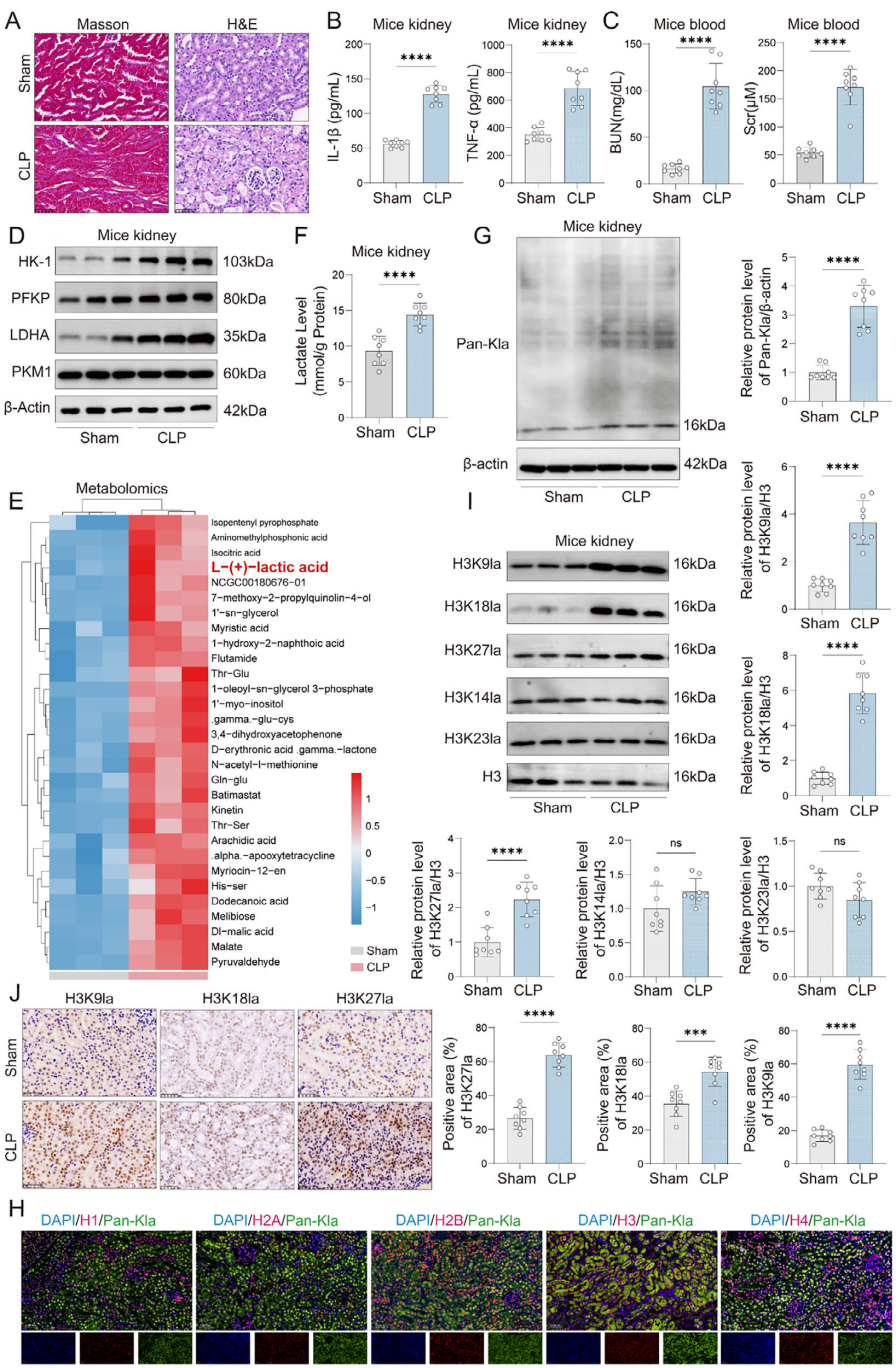
The levels of lactate, lysine lactylation (Kla), H3K9la, H3K18la and H3K27la were increased in CLP mice. (A): Representative microscopic images display haematoxylin and eosin (HE) and Masson's trichrome staining of renal tissue from the sham and CLP groups. Compared to the sham group, CLP mice exhibited more severe tubular swelling and damage. (Scale Bar:50 µm, n = 8). (B): Levels of the inflammatory mediators IL‐1β and TNF‐α in the Sham and CLP groups. (n = 8) (C): Levels of renal injury markers BUN and Scr in the Sham and CLP groups. (n = 8). (D): Western blot revealed increased expression of glycolysis‐related rate‐limiting enzymes HK‐1, PFKP, and LDHA in the CLP group, with no significant change in PKM1. (n = 8). (E): Metabolite levels in mice kidney tissue were normalized relative to the sham group and displayed in a heatmap, with the color scale indicating relative changes. Results revealed a significant increase in lactate levels in the CLP group compared to the sham group. (F): Lactate levels were determined in renal tissue. Compared to sham group mice, CLP mice exhibited elevated lactate levels. (n = 8). (G): Western blot revealed a significant increase in Pan‐Kla levels in CLP mice. (n = 8). H: Pan‐Kla and H1, H2A, H2B, H3, and H4 double immunofluorescence staining in kidney tissue from kidney of CLP mice. Results indicate that Pan‐Kla is predominantly enriched on H3 in CLP group mice. (n = 8). (I): Western blot revealed significantly elevated levels of H3K9la, H3K18la, and H3K27la proteins in CLP group mice compared to sham group mice; whereas H3K14la and H3K23la showed no discernible alterations in CLP group mice. (n = 8). (J): Representative immunohistochemical (IHC) staining for H3K9la, H3K18la and H3K27la was performed on renal tissue sections from sham and CLP mice. Results indicate increased levels of H3K9la, H3K18la, and H3K27la proteins in the CLP group (Scale Bar:50 µm, n = 8). ^*^ *P* < 0.05, ^**^ *P* < 0.01, ^***^ *P* < 0.001, and ^****^ *P* < 0.0001.

To further elucidate the pathogenesis of SA‐AKI in vitro, we established a cellular model of SA‐AKI using lipopolysaccharide (LPS)‐induced HK‐2 cells based on previous research [28, 31]. As the LPS stimulation time increased, Pan‐Kla and H3K9/18/27la reached their peak levels at 12 h (Figure S1B,C). Therefore, we subsequently found that 12 h of LPS induced an increase in lactate and employed a 12‐hour LPS induction as the detection time point (Figure S1D). We found via H1/H2A/H2B/H3/H4 immunoprecipitation (IP) that H3 lactylation was most significantly upregulated in LPS‐treated HK‐2 cells, similar to that in CLP mice (Figure S1E). Advanced results indicated that compared to the control group, LPS treatment increased cellular lactate, Pan‐Kla, and H3K9/18/27la levels (Figure S1F). Furthermore, cell death increased following LPS treatment (Figure S1G,H).

### 2.2 Inhibiting Lactate Production Can Decrease H3K9/18/27la and Cell Death

To further elucidate the role of histone lactylation in SA‐AKI, as agents controlling endogenous lactate production, oxamate (Oxa) and dichloroacetate (DCA) can specifically inhibit the activity of LDHA and PDK, thereby suppressing lactate generation and cellular lactylation [32]. We found that treatment with Oxa or DCA significantly reduced Pan‐Kla, H3K9/18/27la, and lactate levels in the kidneys of CLP mice (Figure 2A–C and Figure S2A–C). Further data indicated that the use of Oxa or DCA alleviated tubular swelling and degeneration caused by SA‐AKI while reducing the levels of inflammatory mediators and renal injury markers within the renal tissue (Figure 2A,D,E and Figure S2A,D,E). We also observed a similar effect in LPS‐induced HK‐2 cells; treatment with Oxa or DCA reduced the LPS‐induced increases in lactate, Pan‐Kla, and H3K9/18/27la, and mitigated the LPS‐induced elevation of LDH and cell death (Figure 2F—I and Figure S2F–I). Next, we increased lactylation levels by the exogenous addition of sodium lactate (NaLa). The results demonstrated that exogenously added NaLa significantly elevated Pan‐Kla and H3K9/18/27la levels in mouse kidneys (Figure S3A,D). Furthermore, it exacerbated IL‐1β, TNF‐α and Scr, BUN concentrations in CLP‐treated mice, while intensifying pathological alterations in renal tubules (Figure S3B,C). Similarly, in HK‐2 cells, NaLa significantly increased Pan‐Kla and H3K9/18/27la levels and exacerbated LPS‐induced lactate dehydrogenase (LDH) release and cell death (Figure S3E–G). To investigate the relationship between H3K9/18/27la and cell death, we introduced single point mutations in H3 by converting lysine residues at positions 9 (K9R), 18 (K18R), and 27 (K27R) into arginine, thereby simulating a delactylated state. The results demonstrated that cells transfected with the K9R/K18R/K27R plasmids exhibited increased resistance to LPS‐induced cell death and LDH release compared to wild‐type cells (Figure S4).

**FIGURE 2:**
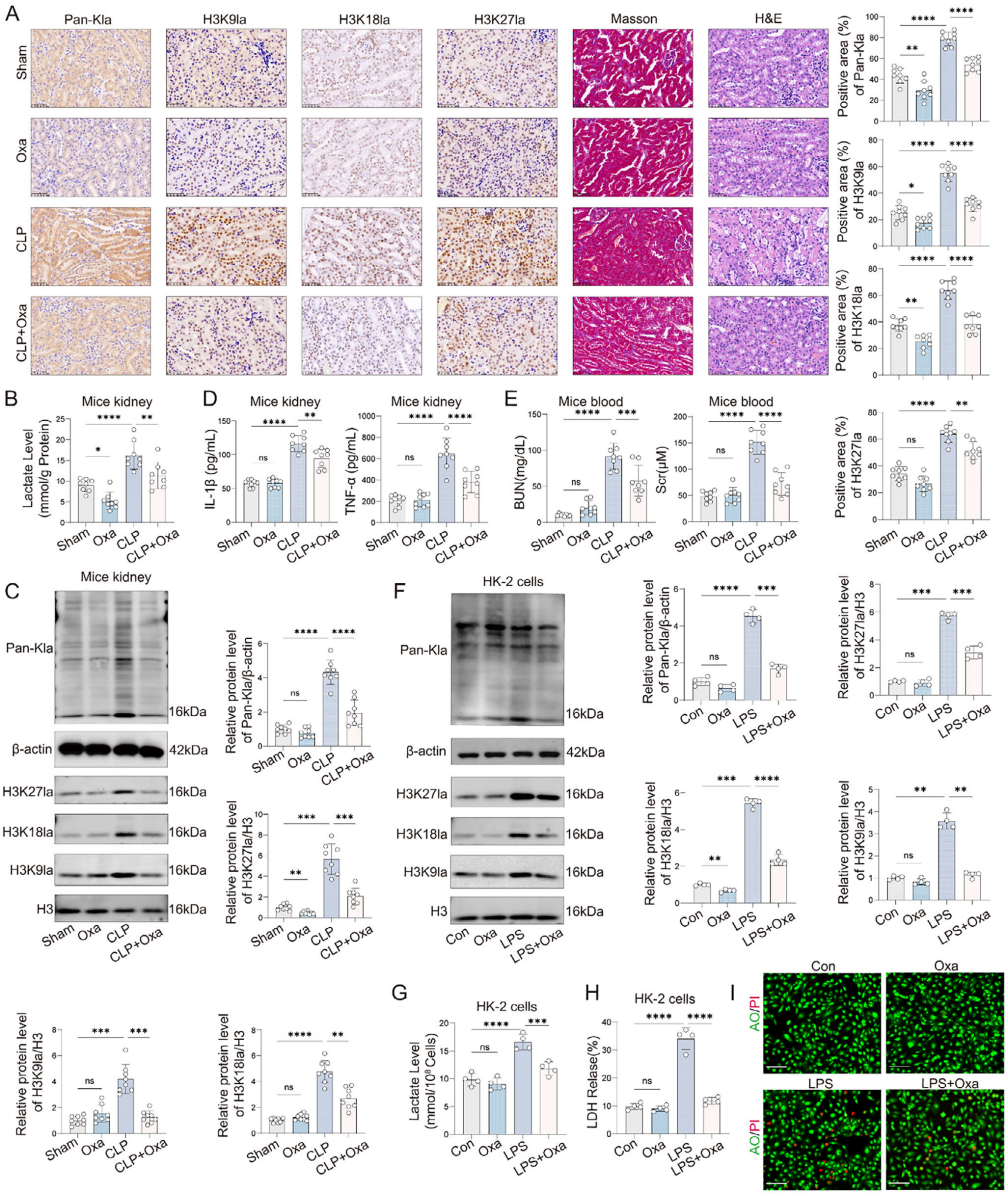
Inhibiting lactate production can decrease H3K9/18/27la and cell death. (A): Representative immunohistochemical (IHC) staining for Pan‐Kla, H3K9la, H3K18la and H3K27la was performed on renal tissue sections from sham, Oxa, CLP, CLP+Oxa mice. Compared with CLP mice, the CLP+Oxa group exhibited lower level of Pan‐Kla, H3K9la, H3K18la and H3K27la. And representative microscopic images display haematoxylin and eosin (HE) and Masson's trichrome staining of renal tissue from the sham, Oxa, CLP, CLP+Oxa mice. Compared to the CLP group, CLP+Oxa mice exhibited milder tubular swelling and damage. (Scale Bar:50 µm, n = 8). (B): Lactate levels were determined in renal tissue. Compared to CLP group mice, CLP+Oxa mice exhibited decreased lactate levels. (n = 8). (C): Western blot revealed that compared to CLP mice, the levels of Pan‐Kla, H3K27la, H3K18la, and H3K9la were significantly reduced in CLP+Oxa mice. (n = 8). (D): Levels of the inflammatory mediators IL‐1β and TNF‐α in the Sham, Oxa, CLP and CLP+Oxa groups. (n = 8). (E): Levels of renal injury markers BUN and Scr in the in the Sham, Oxa, CLP and CLP+Oxa groups. (n = 8). (F): Western blot revealed that compared to LPS treated HK‐2 cells, the levels of Pan‐Kla, H3K27la, H3K18la, and H3K9la were significantly reduced in LPS+Oxa group. (n = 4). (G): Lactate levels were determined in HK‐2 cells. Compared to LPS group, LPS+Oxa mice exhibited decreased lactate levels. (n = 4). (H): LDH release was employed to assess the extent of cell death. Compared to the LPS group, the LPS+Oxa group exhibited lower LDH release in HK‐2 cells. (n = 4). (I): Representative PI staining images. Compared to the LPS group, the LPS+Oxa group exhibited reduced cell death (red indicates dead cells, green indicates viable cells). (Scale Bar:100 µm, n = 4) ^*^ *P* < 0.05, ^**^ *P* < 0.01, ^***^ *P* < 0.001, and ^****^ *P* < 0.0001.

To further elucidate the predominant form of cell death in SA‐AKI, RNA sequencing revealed significant enrichment of pathways associated with apoptosis, necrosis, and pyroptosis (Figure S5A). PANoptosis, a novel form of cell death, encompasses three death pathways, apoptosis, necrosis, and pyroptosis, and plays a crucial role in sepsis [33]. So, we examined PANoptosis‐related markers to determine whether PANoptosis participates in SA‐AKI mice. Western blot confirmed the substantial upregulation of PANoptosis‐related proteins in CLP mice and LPS‐treated HK‐2 cells (Figure S5B,C). We similarly employed Oxa or DCA to inhibit lactylation, with results indicating reduced levels of PANoptosis‐associated proteins in both mouse and cell models (Figure S6). Conversely, exogenous NaLa supplementation increased the expression of PANoptosis‐related proteins (Figure S7). Collectively, these findings suggested that lactylation plays a crucial role in SA‐AKI.

### 2.3 H3K9/18/27la Mediates Cell Death by Promoting Transcription of the NINJ1 in SA‐AKI Model

To further elucidate the mechanism by which H3K9/18/27la modulates cell death, we performed Cut&tag sequencing of H3K9la, H3K18la, and H3K27la in LPS‐induced HK‐2 cells. By integrating RNA‐seq data and PANoptosis‐related gene sets, we identified the candidate gene *Ninj1* (Figure 3A,B). Subsequent chromatin immunoprecipitation‐PCR (CHIP‐PCR) experiments suggested that H3K9/18/27la enriched in the NINJ1 promoter region (Figure S8A). To validate NINJ1 alterations during SA‐AKI, further experiments revealed a significant upregulation of NINJ1 expression in mice kidney and HK‐2 cells (Figure 3C–F and Figure S8B). Furthermore, the human acute kidney injury single‐cell RNA sequencing dataset (GSE199321) also confirmed the upregulation of NINJ1 in renal tubular epithelial cells (Figure S8C–E). Furthermore, transfection of HK‐2 cells with the H3K9/18/27R mutant plasmid led to a decrease in NINJ1 at both mRNA and protein levels, relative to the wild‐type H3 controls, thereby unveiling the transcriptional control exerted by H3K9/18/27la over NINJ1. (Figure S8F–K). Next, ChIP‐qPCR confirmed that LPS treatment significantly increased the enrichment of H3K9la, H3K18la, and H3K27la at the NINJ1 promoter region (Figure S8M). Given that histone lactylation‐mediated chromatin remodeling often cooperates with transcription factors to regulate gene expression, we next sought to identify the transcription factors influencing NINJ1 expression during SA‐AKI. Co‐expression analysis revealed a significant association between NINJ1 and the transcription factor ZHX2 (Figure S8L). Furthermore, ChIP‐reChIP assays confirmed the interaction between H3K9la, H3K18la, H3K27la, and ZHX2 (Figure S8N), suggesting they may jointly regulate NINJ1 gene expression.

**FIGURE 3:**
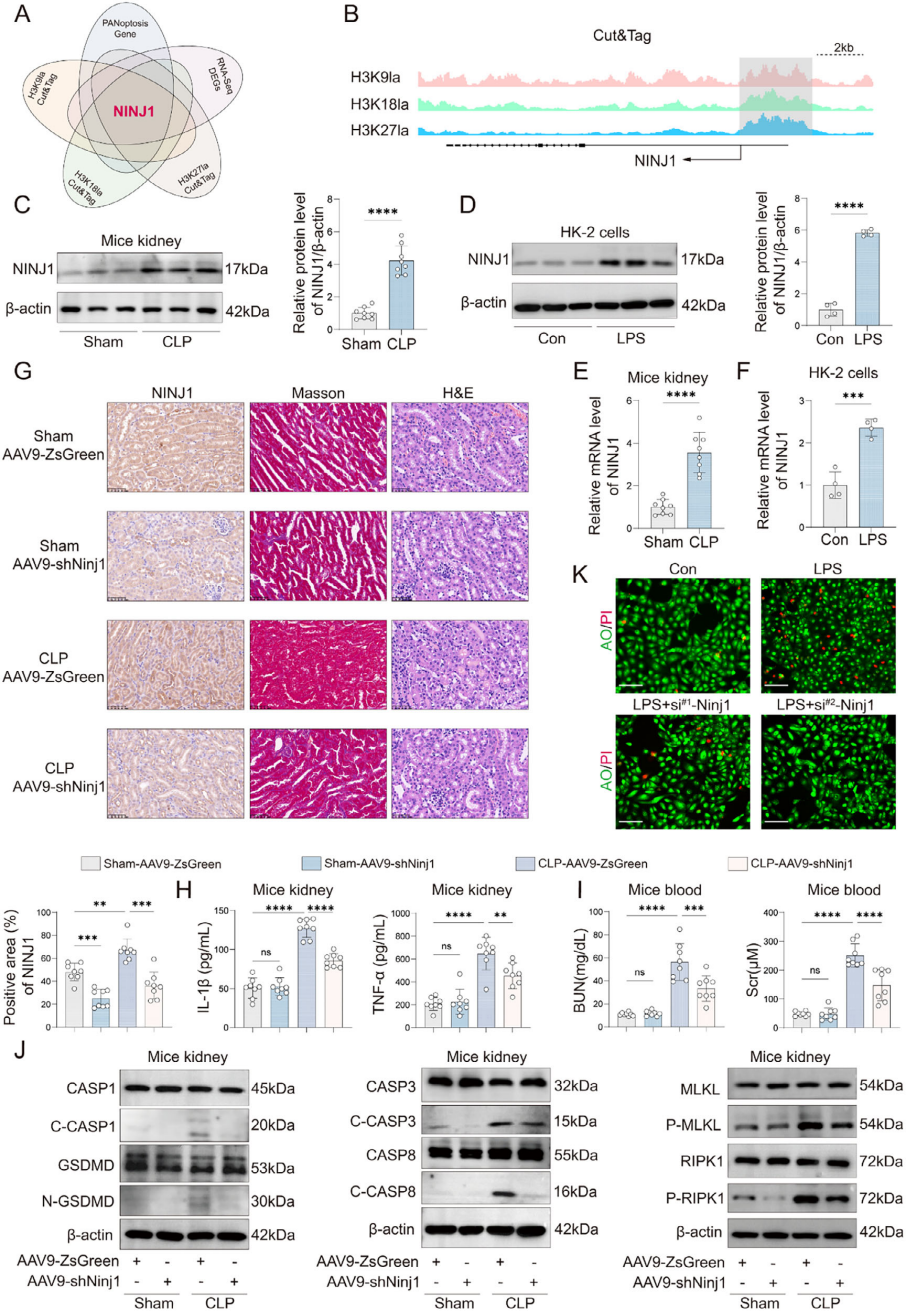
H3K9/18/27la mediates cell death by promoting transcription of the NINJ1 gene in SA‐AKI model. (A): Integrated analysis of RAN‐seq, H3K9la‐Cuttag, H3K18la‐Cuttag, H3K27la‐Cuttag, and PANoptosis gene sets. (B): IGV visualization of H3K9la, H3K18la, and H3K27la enrichment levels in the NINJ1 promoter region. (C): Western blot revealed a significant increase in NINJ levels in CLP mice. (n = 8). (D): Western blot revealed a significant increase in NINJ levels in LPS treated HK‐2 cells. (n = 4). (E): qPCR assay indicated increased NINJ1 mRNA levels in sham and CLP group mice. (n = 8). (F): qPCR assay indicated increased NINJ1 mRNA levels in Con and LPS treated HK‐2 cells. (n = 4). (G): Representative microscopic images display haematoxylin and eosin (HE), Masson's trichrome staining and immunohistochemical staining for NINJ1 of renal tissue from the AAV9‐ZsGreen, AAV‐shNinj1, CLP+AAV9‐ZsGreen, CLP+ AAV‐shNinj1. Compared to the CLP+AAV9‐ZsGreen group, CLP+ AAV‐shNinj1 mice exhibited milder tubular swelling and damage, and lower NINJ1 protein level (Scale Bar:50 µm, n = 8). (H): Levels of the inflammatory mediators IL‐1β and TNF‐α in the AAV9‐ZsGreen, AAV‐shNinj1, CLP+AAV9‐ZsGreen, CLP+ AAV‐shNinj1 groups. (n = 8). (I): Levels of renal injury markers BUN and Scr in the AAV9‐ZsGreen, AAV‐shNinj1, CLP+AAV9‐ZsGreen, CLP+ AAV‐shNinj1 groups. (n = 8). (J): Western blot revealed that compared to CLP+AAV9‐ZsGreen group mice, the levels of PANoptosis related protein (C‐CASP1, N‐GSDMD, C‐CASP3, C‐CASP8, P‐MLKL, P‐RIPK1) were significantly reduced in CLP+ AAV‐shNinj1 group. (n = 8). (K): Representative PI staining images. Compared to the LPS group, the LPS+si^#1^‐Ninj1 and LPS+si^#2^‐Ninj1 group exhibited reduced cell death (red indicates dead cells, green indicates viable cells). (Scale Bar:100 µm, n = 4) ^*^ *P* < 0.05, ^**^ *P* < 0.01, ^***^ *P* < 0.001, and ^****^ *P* < 0.0001.

To investigate the role of NINJ1 in SA‐AKI, we used an adeno‐associated virus 9 (AAV9) to induce NINJ1 knockdown (Figure S9A,B). Moreover, NINJ1 knockdown significantly ameliorated renal pathological alterations, IL‐1β, TNF‐α and Scr, BUN in SA‐AKI mice (Figure 3G–I). In addition, PANoptosis‐associated protein levels were decreased following NINJ1 knockdown (Figure 3J). Similarly, in HK‐2 cells, NINJ knockdown via small interference (si)‐RNA significantly reduced NINJ1 expression (Figure S9C,D), cell death (Figure 3K) and LDH release and decreased the expression of PANoptosis‐associated proteins (Figure S9E,F). In summary, these findings indicated that lactate mediates H3K9/18/27la‐induced PANoptosis by upregulating NINJ1 expression in SA‐AKI.

### 2.4 NINJ1‐K111 Lactylation Promotes its Membrane Localization and Cell Death

In addition to histone lactylation, numerous non‐histone proteins have been shown to undergo lactylation and play significant roles in sepsis [18]. We confirmed the regulation of NINJ1 transcription by H3K9/18/27la; however, post‐translational modifications occurring in NINJ1 remain unknown. Preliminary IP and immunofluorescence co‐localization results suggested that NINJ1 underwent lactylation (Figure 4A–C). Given that NINJ1 possesses only seven lysine residues, we used molecular docking validation to identify potential lactylation sites at lysines 111 (K111la) and 114 (K114la), which are highly conserved across species (Figure 4D). To further validate these sites, we identified individual mutations (lysine to arginine) at K111 and K114 of NINJ1. Subsequent results demonstrated that, compared to the NINJ1 wild‐type, NINJ1 K111R exhibited a lower level of lactylation, while NINJ1 K114R showed a lesser reduction in lactylation (Figure 4E,F), suggesting that NINJ1 K111 may have undergone greater lactylation than K114. Notably, recent research by Wu et al. revealed that NINJ1 K111 and NINJ1 K114 influence plasma membrane localization and cell death [34]. This study provides valuable insights, leading us to hypothesize that K111la and K114la of NINJ1 may affect plasma membrane localization and cell death. To validate the effects of K111la and K114la on NINJ1 plasma membrane localization, we isolated the membrane and cytoplasmic proteins. Results indicated that LPS treatment of HK‐2 cells enhanced NINJ1 plasma membrane expression levels, that both Oxa treatment and NINJ1 K111R inhibited the LPS‐induced increase in NINJ1 plasma membrane expression (Figure 4G–I). Similarly, the use of an NINJ1 plasmid carrying green fluorescent protein (GFP) yielded identical results (Figure 4J). Further results indicated that NINJ1 K111R and K114R significantly reduced LPS‐induced LDH release and cell death (Figure 4K,L). These findings suggest that NINJ1 is not only transcriptionally regulated by H3K9/18/27la, NINJ1‐K111la, but also promotes membrane localization and its capacity to mediate cell death.

**FIGURE 4:**
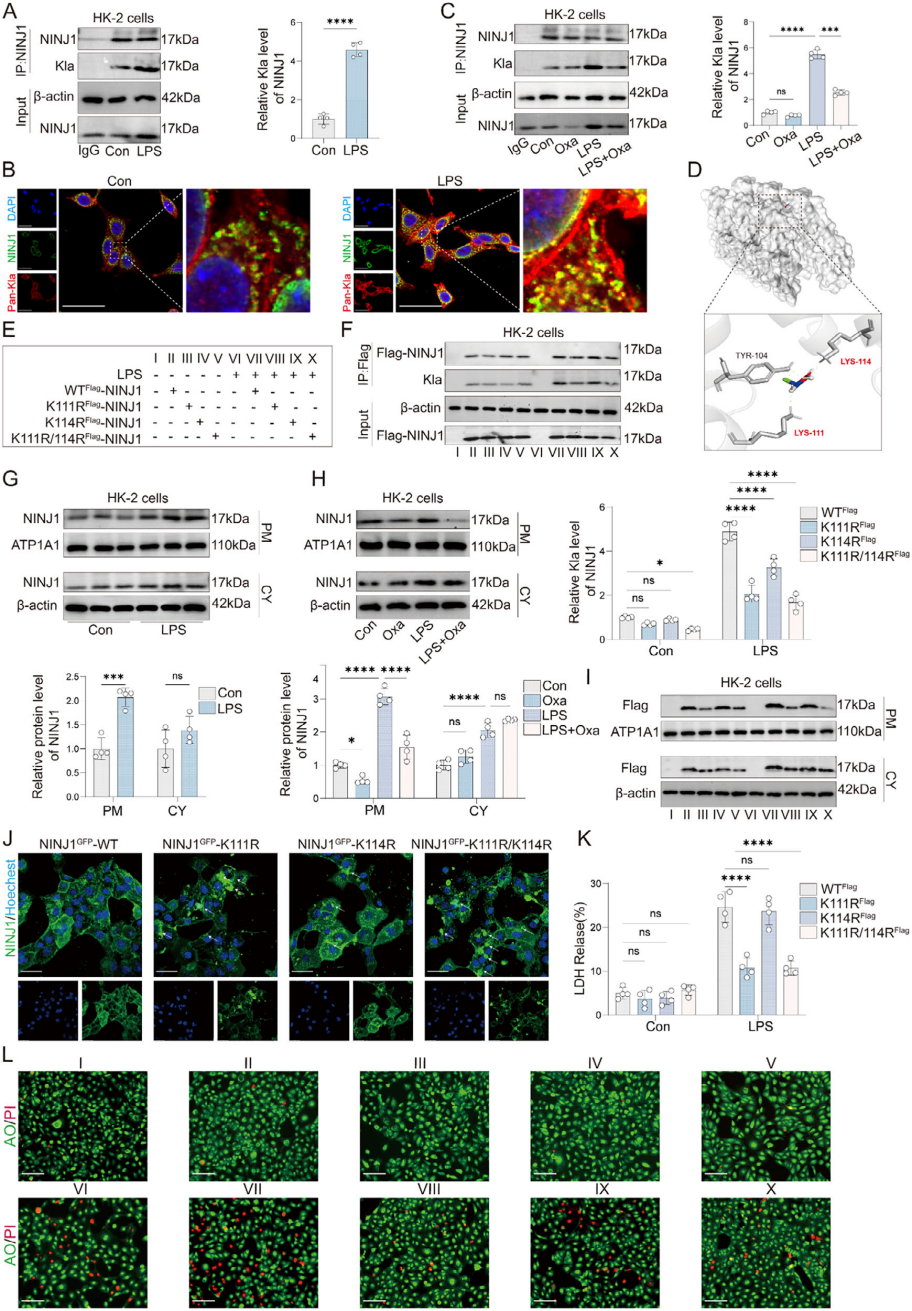
NINJ1‐K111 lactylation promotes its membrane localization and cell death. (A): IP assay revealed a marked increase of NINJ1 lactylation in HK‐2 cells following LPS treatment. (n = 4). (B): Representative immunofluorescence images of NINJ1 and Pan‐Kla from Con and LPS‐treated HK‐2 cells, with blue indicating cell nuclei, green used to visualize NINJ1 and red used to visualize Pan‐Kla (Scale Bar:50 µm, n = 4). (C): IP assay revealed significantly reduced NINJ1 lactylation in the LPS+Oxa group compared to the LPS group in HK‐2 cells. (n = 4). (D): Molecular docking revealed potential sites for lactylation of NINJ1: lysine 111(K111) and lysine 114 (K114). (E): Detailed grouping information. WT^Flag^‐NINJ1, K111R^Flag^‐NINJ1, K114R^Flag^‐NINJ1, and K111R/K114R^Flag^‐NINJ1 were transfected into control and LPS‐treated HK‐2 cells respectively. (F): IP assay revealed that in LPS‐treated HK‐2 cells, K111R ^Flag^‐NINJ1 and K111R/K114R ^Flag^‐NINJ1 group exhibited lower levels of lactylation compared to WT^Flag^ ‐NINJ1 group. (n = 4). (G): Following the separation of plasma membrane (PM) proteins from cytoplasmic (CY) proteins, LPS treatment was shown to increase the membrane‐bound content of NINJ1. (n = 4). (H): Following the separation of membrane proteins from cytoplasmic proteins, the LPS+Oxa group exhibited reduced NINJ1 content in the cell membrane compared to the LPS group. (n = 4). (I): Following the separation of membrane proteins from cytoplasmic proteins, the K111R ^Flag^‐NINJ1 and K111R/K114R ^Flag^‐NINJ1 group exhibited reduced NINJ1 content in the cell membrane compared to the WT ^Flag^ ‐NINJ1 group in LPS treated HK‐2 cells. (n = 4). (J): Representative immunofluorescence images from WT^GFP^‐NINJ1, K111R^GFP^‐NINJ1, K114R^GFP^‐NINJ1, and K111R/K114R^GFP^‐NINJ1. These indicate that K111R^GFP^‐NINJ1 and K111R/K114R^GFP^‐NINJ1 exhibits increased cytoplasmic localization compared to WT‐NINJ1. The arrow indicates NINJ1 accumulated within the cytoplasm. (Scale Bar:50 µm, n = 4). (K): LDH release was employed to assess the extent of cell death. Compared to the WT ^Flag^ ‐NINJ1 group, the K111R ^Flag^‐NINJ1 and K111R/K114R ^Flag^‐NINJ1group exhibited lower LDH release in LPS treated HK‐2 cells. (n = 4). (L): Representative PI staining images. Compared to the WT ^Flag^ ‐NINJ1 group, the K111R ^Flag^‐NINJ1 and K111R/K114R ^Flag^‐NINJ1 group exhibited reduced cell death in LPS treated HK‐2 cells (red indicates dead cells, green indicates viable cells). (Scale Bar:100 µm, n = 4) ^*^ *P* < 0.05, ^**^ *P* < 0.01, ^***^ *P* < 0.001, and ^****^ *P* < 0.0001.

### 2.5 DDX3x is a Potential Delactylase of H3K9/18/27la

The lactylation of histones is often regulated by multiple lactyltransferase, with lactyl‐coA‐dependent types, including P300/ CREB Binding Protein (CBP), histone acetyltransferase (GCN5), MYST (e.g., MOZ, MOF, and TIP60), and HBO1 [35], and lactate‐dependent types, including alanyl‐tRNA synthetase (AARS) 1/2; delactylases primarily comprise Histone Deacetylase (HDAC) 1–3 and Sirtuins (SIRT)1‐3 [36]. Despite some progress in research on delactylases, the extensive influence of HDACs and SIRTs on histone modifications complicates drug development. Consequently, there remains a lack of delactylases capable of specifically regulating lactylation. To identify the delactylases for H3K9/18/27la in SA‐AKI, we performed mass spectrometry (MS) analysis after enrichment using H3K9la, H3K18la, and H3K27la antibodies in LPS‐induced HK‐2 cells. Upon further analysis of the MS results, we observed a significant enrichment of RNA‐binding proteins on H3K9/18/27la (Figure S10A), consistent with previous reports [37]. Among these RNA‐binding proteins, we confirmed that DDX3x (Figure 5A,B), a highly conserved RNA helicase, can interact with H3K9/18/27la through co‐immunoprecipitation (CO‐IP) and GST pull‐down assays (Figure 5C,D). To further validate the potential role of DDX3x to histone lactylation, we simulated the lactylation system in vitro, demonstrated that DDX3x removed H3K9/18/27 lactylation in a concentration‐dependent manner (Figure 5E,F). Dot blotting confirmed the ability of DDX3x to inhibit H3K9/18/27 lactylation (Figure 5G). These findings suggest that DDX3x may functions as a delactylase. To investigate the role of DDX3x in SA‐AKI, our preliminary data indicated reduced DDX3x levels in CLP‐treated mouse kidneys and LPS‐induced HK‐2 cells (Figure S10B–F). On the other hand, the dataset (GSE199321) also confirmed the downregulation of DDX3x during acute kidney injury (Figure S10G). We further overexpressed and knocked down DDX3x in HK‐2 cells. The results revealed that si‐RNA targeting DDX3x (si‐DDX3x) led to an increase in H3K9/18/27la, whereas DDX3x overexpression attenuated the LPS‐induced elevation of H3K9/18/27la (Figure 5H,I). To further validate the active sites of DDX3x as a delactylase, we identified the N537 and K418 residues in DDX3x as potential key positions for histone binding using MS and molecular docking (Figure 5A). To further clarify this, we constructed DDX3x^N537R/K418Q^ mutant (MUT‐DDX3x) plasmids, and molecular docking revealed no significant alterations to the DDX3x structure following the mutation (Figure S11A). The results indicated that compared to the wild‐type DDX3x, MUT‐DDX3x exhibited significantly reduced binding to H3K9/18/27la (Figure S11B). And isothermal titration calorimetry (ITC) demonstrated that MUT‐DDX3x exhibited markedly reduced binding affinity for histone H3 compared with WT‐DDX3x (Figure S11C,D). Furthermore, in vitro, the MUT‐DDX3x recombinant protein demonstrated weaker delactylase activity compared to the WT‐DDX3x recombinant protein (Figure S11E), whereas in LPS‐treated HK‐2 cells, the delactylase activity of MUT‐DDX3x was diminished relative to that of wild‐type DDX3x (Figure S11F).

**FIGURE 5:**
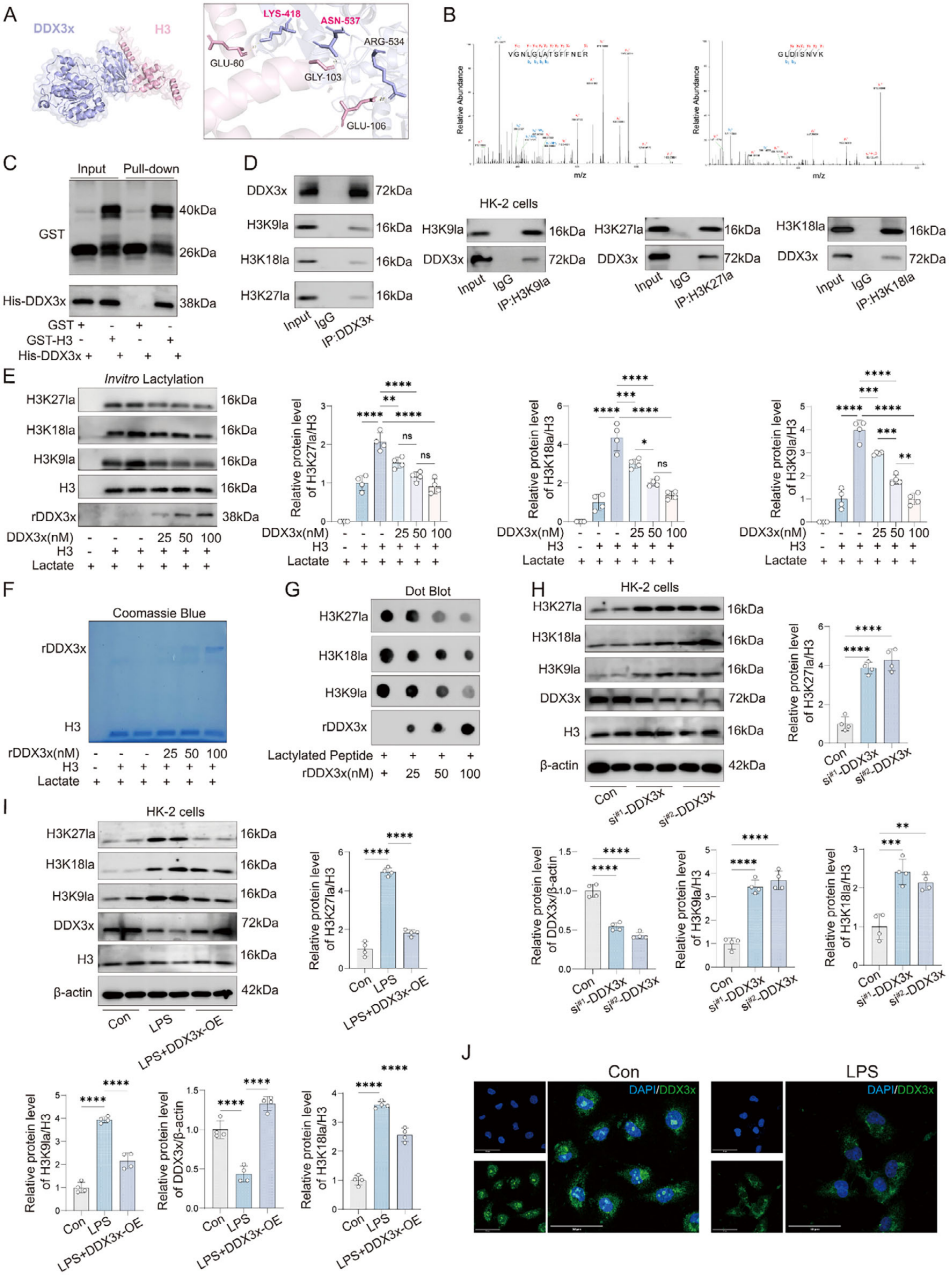
DDX3x is a novel delactylase of H3K9/18/27la. (A): Molecular docking illustrates the binding state of DDX3x with H3. (B): Representative peptides of DDX3x obtained by IP using H3K9la, H3K18la, and H3K27la antibodies. (C): GST pull‐down analysis demonstrates the interaction between DDX3x and H3 in vitro. (n = 4). (D): CO‐IP assays indicated that DDX3x interact with H3K9la, H3K18la, and H3K27la in HK‐2 cells. (n = 4). (E): An in vitro lactylation assay showed that DDX3x served as a delactylase to decrease lactate‐induced histone H3 lactylation in a concentration‐dependent manner. (n = 4). (F): Coomassie Blue staining of the in vitro lactate system. (G): The dot blot analysis indicated that DDX3x delactylated the H3K9la, H3K18la, and H3K27la peptides in a concentration‐dependent manner. (n = 4). (H): Western blot revealed a significant increase in H3K9la, H3K18la, and H3K27la levels following si‐RNA target DDX3x (si‐DDX3x) treatment. (n = 4). (I): Western blot revealed that compared to the LPS group following DDX3x overexpression, levels of H3K9la, H3K18la, and H3K27la were significantly reduced in LPS+DDX3x‐OE group. (n = 4). (J): Representative immunofluorescence images from Con and LPS‐treated HK‐2 cells, with blue indicating cell nuclei and green used to visualize DDX3x (Scale Bar:50 µm, n = 4). ^*^ *P* < 0.05, ^**^ *P* < 0.01, ^***^ *P* < 0.001, and ^****^ *P* < 0.0001.

The DDX family comprises RNA‐binding proteins localized in both the cytoplasm and nucleus. Further investigation revealed that in LPS‐treated HK‐2 cells, despite the reduced overall DDX3x levels, a greater proportion was localized in the cytoplasm (Figure 5J and Figure S11G). These findings were consistent with previous observations made using DDX5 [38]. In summary, these results suggest that DDX3x, may be a novel delactylase. Additionally, we also compared the effects of DDX3x, HDAC1–3, and Sirt1–3 on H3K9/18/27la. These results indicated that DDX3x exhibited de‐lactylase activity toward H3K9/18/27la comparable to that of the previously reported de‐lactylases HDAC1–3 and SIRT1–3, further supporting the identification of DDX3x as a novel de‐lactylase. (Figure S11H).

### 2.6 DDX3x Mediates PANoptosis by Regulating NINJ1 Transcription via H3K9la, H3K18la, and H3K27la

Having previously demonstrated that H3K9la, H3K18la, and H3K27la mediate PANoptosis by regulating NINJ1 transcription, we investigated whether DDX3x influences PANoptosis via H3K9/18/27la by regulating NINJ1 transcription. First, we generated DDX3x tubular epithelial cell‐specific overexpressing mice (DDX3x^f/f Cre+^) by crossing Cre mice carrying the Cadherin 16 promoter with DDX3x‐Flox homozygote mice (DDX3x^f/f^), which showed increased DDX3x expression in renal tubular cells (Figure 6A–C). Further data demonstrated that, compared to DDX3x^f/f^ mice, DDX3x^f/f Cre+^ mice exhibited significantly reduced NINJ1 elevation in SA‐AKI (Figure 6B,D), alongside improved SA‐AKI lesion severity, IL‐1β, TNF‐α, Scr, and BUN levels (Figure 6E–G), whereas PANoptosis protein expression was also diminished (Figure 6H). In HK‐2 cells, overexpression of DDX3x significantly reduced the LPS‐induced elevation of NINJ1 (Figure S12A–C) and inhibited NINJ1‐mediated increases in LDH release (Figure S12D), cell death (Figure S12E), and PANoptosis (Figure S12F). Conversely, si‐DDX3x in HK‐2 cells elevated NINJ1 levels (Figure S13A–C) and enhanced LDH release (Figure S13D), cell death (Figure S13E), and PANoptosis‐associated proteins (Figure S13F). To further validate that cell death induced by DDX3x siRNA was mediated by NINJ1, our data additionally demonstrated that LDH release, cell death, and PANoptosis‐associated proteins increased following DDX3x knockdown, which can be alleviated by si‐NINJ1 (Figure S14). Additionally, we observed that compared with WT‐DDX3x, MUT‐DDX3x attenuated the protective effects of wild‐type DDX3x against LDH release, cell death, and PANoptosis in LPS‐induced HK‐2 cells (Figure S15).

**FIGURE 6:**
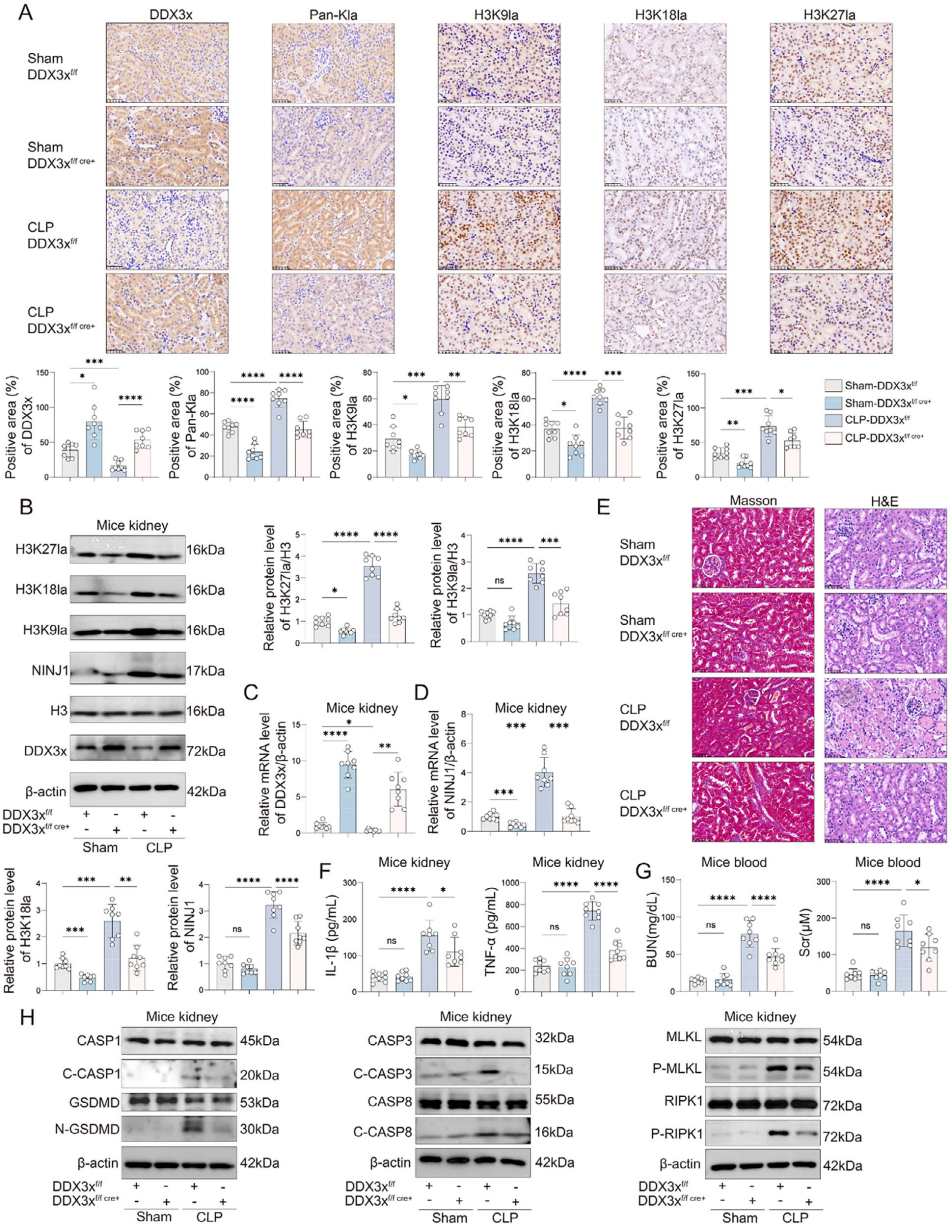
DDX3x mediates PANoptosis by regulating NINJ1 transcription via H3K9, H3K18, and H3K27la. (A): Representative immunohistochemical (IHC) staining for DDX3x, Pan‐Kla, H3K9la, H3K18la, and H3K27la was performed on renal tissue sections from sham‐DDX3x^f/f^, sham‐DDX3x^f/f Cre+^, CLP‐DDX3x^f/f^, CLP‐DDX3x^f/f Cre+^ mice. Compared with CLP‐DDX3x^f/f^, the CLP‐DDX3x^f/f Cre+^ group exhibited increased level of DDX3x, and lower level of Pan‐Kla, H3K9la, H3K18la, and H3K27la (Scale Bar:50 µm, n = 8). (B): Western blot revealed that compared to CLP‐DDX3x^f/f^, the levels of Pan‐Kla, H3K27la, H3K18la, H3K9la, and NINJ1 were significantly reduced, with DDX3x level increased in CLP‐DDX3x^f/f Cre+^ group. (n = 8). (C): qPCR assay indicated increased DDX3x mRNA levels in sham‐DDX3x^f/f^, sham‐DDX3x^f/f Cre+^, CLP‐DDX3xf/f, CLP‐DDX3x^f/f Cre+^ mice. (n = 8). (D): qPCR assay indicated increased NINJ1 mRNA levels in sham‐DDX3x^f/f^, sham‐DDX3x^f/f Cre+^, CLP‐DDX3x^f/f^, CLP‐DDX3x^f/f Cre+^ mice. (n = 8). (E): Representative microscopic images display haematoxylin and eosin (HE) and Masson's trichrome staining of renal tissue from the sham‐DDX3x^f/f^, sham‐DDX3x^f/f Cre+^, CLP‐DDX3x^f/f^, CLP‐DDX3x^f/f Cre+^ mice. Compared to the CLP‐DDX3x^f/f^ group, CLP‐DDX3x^f/f Cre+^ mice exhibited milder tubular swelling and damage. (Scale Bar:50 µm, n = 8). (F): Levels of the inflammatory mediators IL‐1β and TNF‐α in the sham‐DDX3x^f/f^, sham‐DDX3x^f/f Cre+^, CLP‐DDX3x^f/f^, CLP‐DDX3x^f/f Cre+^ mice. (n = 8). (G): Levels of renal injury markers BUN and Scr in the in the sham‐DDX3x^f/f^, sham‐DDX3x^f/f Cre+^, CLP‐DDX3x^f/f^, CLP‐DDX3x^f/f Cre+^ mice. (n = 8). (H): Western blot revealed that compared to CLP‐DDX3x^f/f^ group mice, the levels of PANoptosis related protein (C‐CASP1, N‐GSDMD, C‐CASP3, C‐CASP8, P‐MLKL, P‐RIPK1) were significantly reduced in CLP‐DDX3x^f/f Cre+^ mice. (n = 8) ^*^ *P* < 0.05, ^**^ *P* < 0.01, ^***^ *P* < 0.001, and ^****^ *P*<0.0001.

### 2.7 Ode Alleviates SA‐AKI by Activating the Delactylase Activity of DDX3x

Given the crucial role of DDX3x in NINJ1‐mediated PANoptosis in SA‐AKI via H3K9/18/27la, we conducted virtual drug screening using the HY‐L001P MCE Bioactive Compound Library Plus library database based on molecular docking. The results indicated potential binding interactions between odetiglucan (Ode) and DDX3x, and further molecular dynamics simulations revealed the binding mode between Ode and DDX3x (Figure S16A,B). ITC confirmed binding of DDX3x‐Ode in vitro (Figure S16C). To better evaluate the effect of Ode on DDX3x and lactylation, our initial findings in an in vitro lactylation system indicated that Ode decreased the level of H3K9/18/27la (Figure S16D). These findings suggested that Ode may modulates NINJ1 via DDX3x, thereby alleviating SA‐AKI. To validate this hypothesis, we first observed that in CLP mice, Ode significantly reduced H3K9/18/27la levels in renal tissue and suppressed NINJ1 expression (Figure 7A,B). Concurrently, Ode treatment markedly improved renal pathological alterations in CLP mice, reducing levels of IL‐1β, TNF‐α, Scr, BUN, and PANoptosis‐associated proteins (Figure 7D–G). In HK‐2 cells, Ode treatment reduced H3K9/18/27la and NINJ1 levels (Figure 7C and Figure S16E). Similarly, we demonstrated that Ode treatment significantly diminished LPS‐induced NINJ1 expression without change of DDX3x (Figure S16F–H), and reduced LDH release (Figure S16I), cell death (Figure 7H), and PANoptosis‐associated proteins (Figure S16J). Moreover, Ode also improved the 7‐day survival rate of CLP mice (Figure S16K). Notably, to further ascertain that the regulatory effect of Ode on H3K9/18/27la is mediated through DDX3x, subsequent experiments revealed that DDX3x knockdown by siRNA abolished the effect of Ode on H3K9/18/27la (Figure S16L).

**FIGURE 7:**
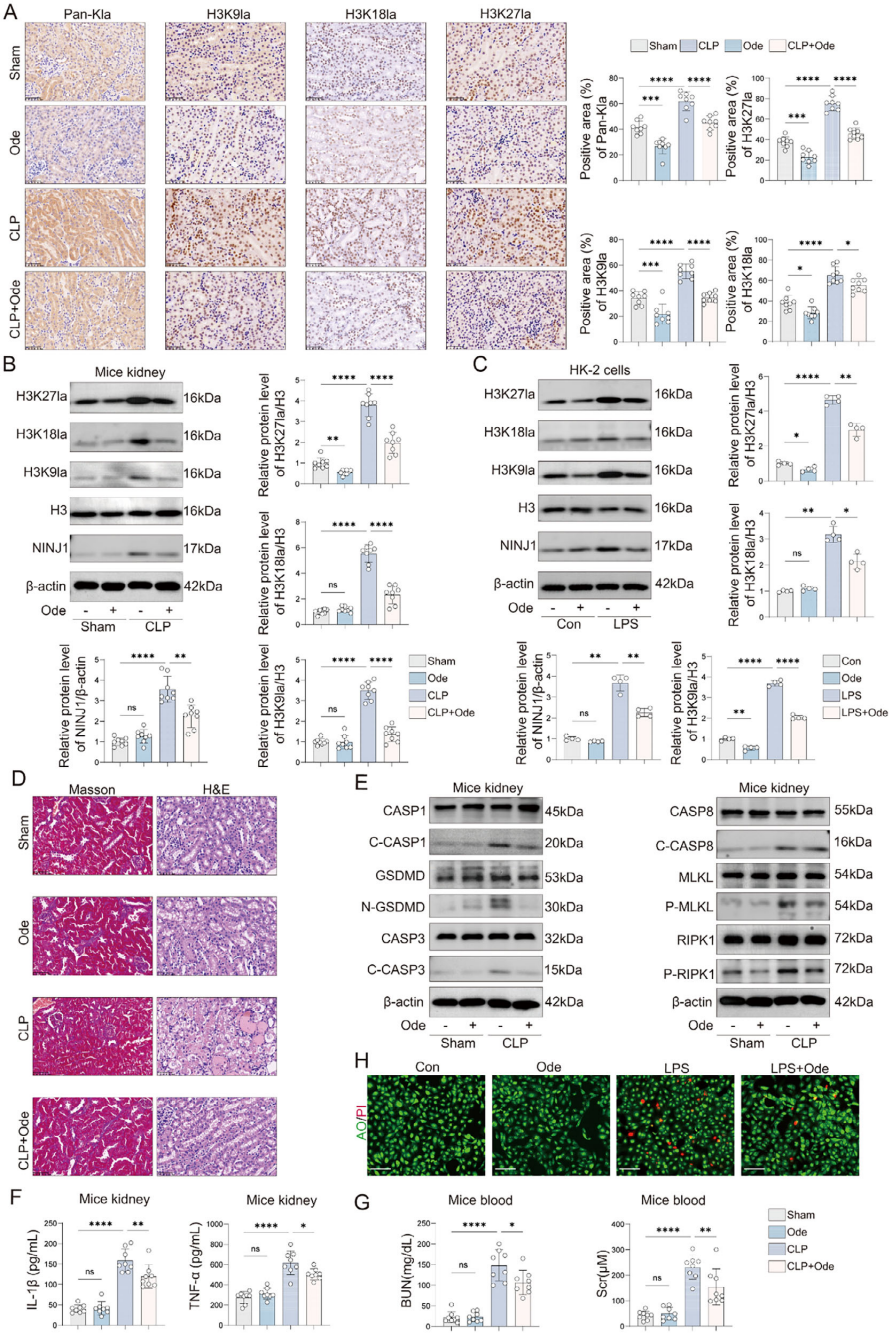
Ode alleviates SA‐AKI by activating the delactylase activity of DDX3x. (A): Representative immunohistochemical (IHC) staining for Pan‐Kla, H3K9la, H3K18la, and H3K27la was performed on renal tissue sections from sham, Ode, CLP, CLP+Ode mice. Compared with CLP, CLP+Ode group exhibited lower level of Pan‐Kla, H3K9la, H3K18la, and H3K27la (Scale Bar:50 µm, n = 8). (B): Western blot revealed that compared to CLP mice, the levels of H3K27la, H3K18la, H3K9la, and NINJ1 were significantly reduced in CLP+Ode group. (n = 8). (C): Western blot revealed that compared to LPS group, the levels of H3K27la, H3K18la, H3K9la, and NINJ1 were significantly reduced in LPS+Ode group in HK‐2 cells. (n = 4). (D): Representative microscopic images display haematoxylin and eosin (HE) and Masson's trichrome staining of renal tissue from sham, Ode, CLP, CLP+Ode mice. Compared to the CLP group, CLP+Ode mice exhibited milder tubular swelling and damage. (Scale Bar:50 µm, n = 8). (E): Western blot revealed that compared to CLP group mice, the levels of PANoptosis related protein (C‐CASP1, N‐GSDMD, C‐CASP3, C‐CASP8, P‐MLKL, P‐RIPK1) were significantly reduced in CLP+Ode mice. (n = 8). (F): Levels of the inflammatory mediators IL‐1β and TNF‐α in sham, Ode, CLP, CLP+Ode mice. (n = 8). (G): Levels of renal injury markers BUN and Scr in the in sham, sham+Ode, CLP, CLP+Ode mice. (n = 8). (H): Representative PI staining images. Compared to the LPS group, LPS+Ode group exhibited reduced cell death in HK‐2 cells (red indicates dead cells, green indicates viable cells). (Scale Bar:100 µm, n = 4) ^*^ *P* < 0.05, ^**^ *P* < 0.01, ^***^ *P* < 0.001, and ^****^ *P* < 0.0001.

In summary, these results demonstrate that Ode may serve as a promising therapeutic agent by targeting the DDX3x‐H3K9/18/27la‐NINJ1‐PANoptosis axis, thereby alleviating or preventing SA‐AKI.

### 2.8 Ode Also Alleviates Lung Injury in CLP Mice

Sepsis is a systemic disease triggered by infection. In addition to SA‐AKI, sepsis‐associated lung injury plays a significant role in mortality among patients with sepsis. Recent studies have demonstrated the relationship between sepsis‐associated lung injury and histone lactylation [39, 40]. According to this evidence, we explored the effect of Ode in modulating H3K9/18/27la via DDX3x in sepsis‐associated lung injury. First, we observed that in CLP mice lung tissue, similar to renal tissue, DDX3x expression was reduced; meanwhile, H3K9/18/27la and NINJ1 expression increased (Figure S17A,B). Following Ode treatment, H3K9/18/27la and NINJ1 levels significantly reduced (Figure S17C–F), and pathological alterations in CLP‐induced lung injury and PANoptosis‐associated proteins were alleviated (Figure S17D,G–I). To further evaluate the safety of Ode, we examined markers of injury in other organs including Cardiac troponin I (cTnT), Brain Natriuretic Peptide (BNP), alanine aminotransferase (ALT) and found that Ode administration caused no significant damage to these organs (Figure S17J–L). Our data demonstrate that enhancing DDX3x delactylase activity via Ode may represent a promising therapeutic strategy for sepsis‐related complications, including sepsis‐associated lung and renal injuries.

## 3 Discussion

In this study, we systematically elucidated the process by which lactate increases H3K9/18/27la, thereby promoting NINJ1 transcription. This process facilitates PANoptosis, ultimately leading to SA‐AKI. Furthermore, DDX3x, a newly identified delactylase, was downregulated in SA‐AKI. By inhibiting H3K9/18/27la, DDX3x subsequently suppresses NINJ1 transcription, thereby inhibiting PANoptosis. Notably, we identified Ode, a drug that activates DDX3x delactylase activity, and evaluated its role in regulating PANoptosis via DDX3x in SA‐AKI through in vitro and in vivo studies.

Extensive research has demonstrated the roles of cellular metabolic reprogramming, oxidative stress, and inflammatory responses in SA‐AKI [28, 41]. As primary cells are affected in SA‐AKI, the death of renal tubular epithelial cells represents a crucial molecular change. PANoptosis, a novel form of cell death, is mediated by the PANoptosome, whose molecular initiation depends on the conformational activation and polymerization assembly of pattern recognition receptors, such as ZBP1 and AIM2 [42]. Recent studies have confirmed that LPS activates AIM2 inflammasomes to promote ASC oligomerization, thereby triggering PANoptosis in LPS induced HK‐2 cells [43]. NINJ1, a novel protein that mediates plasma membrane rupture during cell death, has recently been implicated in the release of damage‐associated molecular patterns (DAMPs) after membrane rupture, thereby inducing PANoptosis [14, 15]. Our findings demonstrated increased level of PANoptosis‐associated proteins and elevated NINJ1 levels in CLP mice kidney tissue, with similar results observed in LPS‐induced HK‐2 cells. Furthermore, AAV9‐shNINJ1exhibited reduced inflammatory levels and PANoptosis‐associated proteins in CLP renal tissue. These findings suggest that inhibition of NINJ1 may represent a potential therapeutic or preventive strategy against SA‐AKI.

Lactate, a serum marker for sepsis, was discovered in 2019 to be capable of substrate‐specific modification of lysine residues in proteins, a process termed lactylation [17]. Histones, especially H3 and H4, participate in chromatin remodeling, and the lactylation of histones can lead to the change of the chromatin accessibility. Recently, mounting evidence has demonstrated the critical role of histone lactylation in sepsis and its complications [40, 44]. Consistent with these studies, our results revealed increased H3K9/18/27la levels in CLP mouse kidneys and LPS‐treated HK‐2 cells. Further analysis demonstrated that H3K9/18/27la have been enriched in the NINJ1 promoter region, suggesting that lactate may promotes NINJ1 transcription via H3K9/18/27la. Moreover, the inhibition of lactate‐mediated H3K9/18/27la using Oxa or DCA significantly reduced NINJ1 transcription levels, thereby alleviating PANoptosis. These findings suggested that lactate promoted PANoptosis in SA‐AKI by regulating NINJ1 transcription via H3K9/18/27la. Notably, beyond H3K9/18/27la‐mediated regulation of NINJ1 transcription, our study also identified NINJ1 K111 and K114 lactylation. Although previous studies have revealed a link between NINJ1‐K111 and ‐K114, and both plasma membrane localization and cell death mediation, the precise mechanism remains elusive [34]. Our findings demonstrate that NINJ1‐K111, the primary lactylation site, significantly mediates the plasma membrane localization of NINJ1, thereby establishing a foundation for NINJ1‐mediated PANoptosis. In summary, lactate simultaneously promotes NINJ1 transcription via H3K9/18/27la and mediates NINJ1 lactylation. This ultimately elevates NINJ1 expression, accelerates its plasma membrane localization, and triggers PANoptosis.

Lactyltransferases and delactylases, as primary regulators of histone lactylation, have been demonstrated to play important roles in common disease processes [45]. Nevertheless, the functional roles and specificities of lactyltransferases and delactylases within the pathological context of SA‐AKI remain poorly defined. Identifying and regulating lactyltransferase and delactylase activities of H3K9/18/27la in SA‐AKI may facilitate the development of promising therapeutic interventions. Several lactylation‐regulating enzymes have been identified, such as p300/CBP, HBO1, AARS1/2, SIRT1‐3, HDAC1‐3 [46, 47, 48]. Further analysis of the MS data identified that DDX3x can interacts with H3K9/18/27la, which was validated by CO‐IP and GST‐pulldown assays. Moreover, in an in vitro lactylation system, DDX3x exhibited concentration‐dependent reduction in H3K9/18/27la cells. As an RNA helicase, DDX3x participates in multiple RNA metabolic processes and is highly conserved in eukaryotes. Previous studies have suggested DDX3x involvement in immune regulation during sepsis [49], while bioinformatics analysis revealed its potential association with PANoptosis in septic patients [27]. However, the specific role and mechanism of action of DDX3x in SA‐AKI remains unclear. In this study, we have for the first time identified DDX3x as a previously unreported delactylase, whose expression is downregulated in CLP mouse kidneys and LPS‐induced HK‐2 cells. Further data revealed that DDX3x^f/f Cre+^‐CLP mice exhibited reduced levels of H3K9/18/27la and NINJ1, along with milder renal injury manifestations compared to DDX3x^fl/fl^ ‐CLP mice. Similar results were observed when DDX3x was overexpressed in LPS‐stimulated HK‐2 cells. Moreover, si‐DDX3x significantly increased the expression of H3K9/18/27la and NINJ1, leading to increased cell death and PANoptosis‐associated protein expression. Furthermore, by integrating MS data with molecular docking, we identified key binding sites for DDX3x at H3K9/18/27la: N537 and K418. Subsequently, we constructed DDX3x^N537R/K418Q^ plasmids. The results demonstrated that compared to wild‐type DDX3x, N537R/K418Q eliminated DDX3x binding to H3K9/18/27la and attenuated the protective effect of DDX3x against LPS‐induced PANoptosis in HK‐2 cells. In summary, these findings demonstrate that DDX3x, a novel delactylase, modulates H3K9/18/27la to inhibit NINJ1 transcription, thereby ameliorating PANoptosis in SA‐AKI.

Owing to a crucial role of DDX3x in SA‐AKI by regulating NINJ1 transcription via H3K9/18/27la and mediating PANoptosis, the specific activation delactylase function of DDX3x holds promise as an effective therapeutic strategy for SA‐AKI. Ode, a novel β‐glucan, serves as a potent immunostimulant. Ode activates innate immune effector cells, triggering a coordinated antitumor immune response. Previous studies have confirmed its therapeutic efficacy against hepatocellular carcinoma [50]; however, its precise role in sepsis remains unclear. In this study, virtual screening and ITC revealed a binding interaction between Ode and DDX3x. Furthermore, in vitro lactylation assays confirmed the activating effect of Ode on DDX3x delactylase activity. Consequently, we administered Ode to LPS‐induced HK‐2 cells and CLP‐induced SA‐AKI in mice. Our findings demonstrated that ode reduced H3K9/18/27la levels, inhibited NINJ1 transcription, and diminished PANoptosis both in vivo and in vitro. We found that by targeting the activation of the delactylase activity of DDX3x, ode may be a promising therapeutic strategy for SA‐AKI.

However, this study has certain limitations. First, we did not collect data from patients with sepsis to validate underlying mechanisms. Future research should investigate the therapeutic efficacy of Ode in patients with SA‐AKI. Second, although this study has demonstrated that Ode ameliorates SA‐AKI by regulating renal tubular epithelial cell death through DDX3x, further studies are required to determine the pharmacokinetics, pharmacodynamics, toxicological profiles and therapeutic window of Ode in the treatment of SA‐AKI, thereby enhancing its clinical translational value. Third, whether ZHX2 serves as the bona fide transcription factor linking H3K9/18/27la to NINJ1 regulation in different disease models and cell types warrants further investigation. Fourth, this study identified the catalytic residues K418 and N537 of DDX3x, but enzymatic kinetic analysis has not been performed; determination of its kinetic parameters, including K_m_, V_max_, and K_cat_. Nevertheless, further exploration of the time‐course delactylation of DDX3x in future work would be valuable.

In summary, our research confirmed that DDX3x—a previously unreported delactylase—modulates H3K9/18/27la, thereby influencing NINJ1 transcription and PANoptosis in SA‐AKI. Ode, a delactylase activator of DDX3x, is a potential therapeutic agent for SA‐AKI.

## 4 Methods

### 4.1 Mouse Model of CLP

Male or female C57BL/6 mice, DDX3x heterozygous (DDX3x^fl/+^) on the genetic background of C57BL/6, and tool mice of C57BL/6 carrying the Cdh16‐Cre (8 weeks old, body weight 20–30 g) were procured from Jiangsu Jicui Biotechnology Co., Ltd. (Nanjing, China). Ethical clearance for animal experiment conducted was granted by the Animal Ethics Committee of Shanghai Yanlu Bio‐Pharmaceutical Co., Ltd (Approval No.: YL20250228‐4). A controlled ambient temperature spanning 22°C–25°C and a regulated 12‐h light‐dark alternated regimen were maintained for animal housing. The CLP mouse model was established as previously described [51]. Following isoflurane anesthesia, the cecum was exposed via a 1 cm midline abdominal incision. The cecum was ligated between the third and fourth lumbar vertebrae using a 4‐0 silk suture and punctured twice using a 25‐gauge needle. A small amount of fecal matter (approximately 1 mm in length) was expelled. Following surgery, the incision was sutured in two layers and recovery was facilitated by a subcutaneous injection of 1 mL sterile 0.9% saline. The mice were then placed on a heating pad for recovery. Sham mice that did not undergo cecal ligation, served as controls. The kidneys and lungs were harvested from the CLP mice at 24 h for subsequent experiments. Twenty‐four hours prior to the commencement of either sham or CLP surgery, a pre‐operative intraperitoneal injection of 750 mg/kg body weight Oxamate (HY‐W013032A, MedChemExpress (MCE), Shanghai, China), or 25 mg/kg body weight DCA (HY‐Y0445A, MCE) was administered, defining the groups as Oxa, CLP+Oxa or DCA, CLP+DCA. Similarly, preoperative intraperitoneal injection of 500 mg/kg body weight NaLa (HY‐B2227B, MCE, China) administered 24 h prior to Sham or CLP surgery was defined as the NaLa and CLP+NaLa groups. The experiments were divided into the following groups (n = 8): Sham, CLP, Oxa, CLP+Oxa, DCA, CLP+DCA, NaLa, and CLP+NaLa. Serum was collected from the peripheral blood of the mice for further analysis.

### 4.2 AAV9 Administration

A recombinant AAV9 vector carrying either the NINJ1 coding sequence (AAV9‐Cdh16‐NINJ1) or the fluorescent reporter ZsGreen (AAV9‐Cdh16‐ZsGreen) under the renal tubular epithelial‐specific Cadherin 16 (Cdh16) promoter was generated by Oberon Biotech (Shanghai, China). Three weeks before sham or CLP surgery, mice received a single tail‐vein injection of the respective vector at a dose of 5 × 10^1^ ^1^ viral genomes (vg) per animal.

### 4.3 Histological Staining and Immunohistochemical (IHC) Analysis

Histological segments prepared from paraffin blocks were first desiccated for a 2 h duration within a 60°C oven, before being cleared with xylene and subsequently passed through a step‐down series of ethyl alcohol concentrations for tissue rehydration. For hematoxylin and eosin (H&E) staining, nuclei were stained with hematoxylin (C0105; Beyotime, Shanghai, China) for 10 min, followed by a 30‐second counterstain of the cytoplasm with eosin. After staining, sections were mounted with neutral balsam, air‐dried, and examined under a light microscope.

Masson's trichrome staining was performed as follows: the cytoplasm and muscle fibers were stained with an acid fuchsin acid mordant. Following treatment with phosphotungstic phosphomolybdic acid, the collagen fibers were stained with aniline blue. After differentiation, dehydration, and mounting, the stained sections exhibited blue collagen fibers, red myofibrils and cytoplasm, and dark purple nuclei.

For immunohistochemical staining, antigen retrieval was performed using phosphate‐buffered saline (PBS; pH 6.0, 10 mM; GK500705, GeneTech, Shanghai, China). Endogenous peroxidase activity was quenched with 3% hydrogen peroxide (H1009, GeneTech), and block with 5% goat serum (G9023, Sigma–Aldrich, St. Louis, MO, USA). The sections were then incubated with primary antibodies of H3K9la (1:200, PTM‐1419RM, PTM‐Bio, Hangzhou, China), H3K18la (1:200, PTM‐1406RM, PTM‐Bio), H3K27la (1:200, PTM‐1428, PTM‐Bio), Pan‐Kla (1:200, PTM‐1429RM, PTM‐Bio), DDX3x (1:200, 11115‐1‐AP, Proteintech, Wuhan, China), NINJ1(1:100, PC‐11105, Abmart, Shanghai, China), followed by incubation with an HRP‐conjugated secondary antibody (GK500705, GeneTech). Color development was achieved using a DAB substrate kit (GK347011; GeneTech). Finally, the sections were cleared in xylene, covered with coverslips, and observed under a light microscope.

#### 4.3.1 Quantitative Analysis of IHC Staining

Immunohistochemical staining intensity was quantified using ImageJ. Images were converted to 8‐bit grayscale and thresholded to highlight positive staining. The stained area and mean grey value were measured, and staining intensity was calculated as the integrated density (sum of pixel values) divided by the stained area.

### 4.4 Cell Culture and Treatment

HK‐2 cells were obtained from Procell (CL‐0109, Procell, Wuhan, China) and maintained in DMEM/F12 (11320033; Thermo Fisher Scientific, Waltham, MA, USA) for subculturing. Cells were treated with 10 µg/ml LPS (L2880, Sigma–Aldrich) diluted in DMEM/F12 for 12 h. The control group was administered equivalent volumes of DMEM/F12 for the same duration. For lactylation inhibition, cells were pretreated with 10 mM of the LDHA inhibitor oxamate (HY‐W013032A, MCE, China) for 3 h, defining the Oxa and LPS + Oxa groups. Similarly, cells were pretreated with 5 mM DCA (HY‐Y0445A; MCE, China) for 3 h, constituting the DCA and LPS + DCA groups. To further investigate the effect of lactate in LPS‐induced PANoptosis, cultured cells were induced with 10 mM NaLa (HY‐B2227B, MCE) or with 10 µg/ml LPS plus 10 mM NaLa. Additionally, Ode (HY‐147295; MCE) was used to activate the delactylase activity of DDX3x, thereby inhibiting H3K18la, H3K27la, and H3K9la expression in the LPS‐treated cells. To determine the optimal concentration for suppressing H3K18la, H3K27la, and H3K9la, HK‐2 cells were co‐treated with 10 µg/mL LPS and Ode at concentrations of 50, 100, 200, and 500 µg/ml for 12 h.

### 4.5 siRNA/Plasmid Transfection

For HK‐2 cell manipulation, Lipofectamine 3000 (L3000015, Thermo Fisher Scientific) was introduced to samples at 70%–80% confluency. The initial step required incorporating 2500 ng of plasmid DNA into the P3000 reagent, subsequent blending with Lipofectamine 3000 yielded transfection complexes that were promptly introduced into the cell culture. Table S1 details the sequences of the targeting siRNAs used in this study.

### 4.6 Quantitative Real‐Time PCR (qPCR)

Gene expression in mouse kidney and lung tissues and in HK‐2 cells was measured by quantitative real‐time PCR (qPCR). Total RNA was isolated using RNA Purification Kit (EZB‐RN4; EZbioscience, Roseville, MN, USA). And a NanoDrop spectrophotometer was employed to determine both the yield and optical density ratios of the isolated RNA. To generate first‐strand cDNA, 1 µg of total RNA underwent reverse transcription utilizing HyperScript III SuperMix (R202‐02; EnzyArtisan, Shanghai, China). qPCR was carried out on an ABI 7500 Real‐Time PCR System (Applied Biosystems, USA) using Universal SYBR Mix (EnzyArtisan). Relative expression was determined by the 2^−ΔΔCt^ method, with β‐actin as the endogenous control. Table S1 list the specific primer sequences utilized in this study.

### 4.7 Western Blot Assay

Protein extracts from mouse tissues or cultured cells were prepared for western blot analysis. The samples were lysed using RIPA buffer (PC101; Epizyme Biotech, Shanghai, China) supplemented with phenylmethylsulfonyl fluoride (PMSF; ST2573, Beyotime). The protein concentration was determined using the bicinchoninic acid (BCA) method (P0009; Beyotime, Shanghai, China). Following the resolution of identical protein quantities (30 µg) via 8%–15% SDS‐PAGE, the separated bands were electroblotted onto polyvinylidene fluoride (PVDF) matrices (ISEQ00010, Millipore, Bedford, MA, USA). Subsequent to the blocking, individual primary antibodies were applied to the target membranes for an overnight duration maintained at 4°C (refer to Table S1 for antibody information). After washing, the membranes were incubated with horseradish peroxidase‐conjugated secondary antibodies (SA00001‐2; SA00001‐1, Proteintech) for 1 h at room temperature. β‐Actin was used as an internal control.

### 4.8 Acridine Orange/ Propidium Iodide (AO/PI) Staining

Treated HK‐2 cell viability and cytotoxicity were evaluated using ViaStain AOPI staining solution (CS2‐0106‐5ML, Revvity, Waltham, MA, USA), which contains acridine orange (AO) and propidium iodide (PI). After treatment, cells were washed twice with PBS and incubated with the staining solution at 37°C for 20 min in the dark. Following a single PBS rinse, cells were immediately imaged under a Zeiss fluorescence microscope. Live cells emitted green fluorescence, whereas dead or membrane‐compromised cells emitted red fluorescence. At least five random fields were captured per sample, and the ratio of PI‐positive cells was quantified using ImageJ.

### 4.9 Immunofluorescence Staining

#### 4.9.1 Immunofluorescence Staining of HK‐2 Cells

Permeabilization of the HK‐2 cells was achieved using Triton X‐100 (0.3%), sequentially followed by a non‐specific blocking step with goat serum (5%) maintained for 1 h at room temperature. Overnight incubation at 4°C was subsequently performed using primary antibodies against DDX3x (1:100; Proteintech, 11115‐1‐AP), Pan‐Kla (1:200; PTM‐Bio, PTM‐1429RM), and NINJ1 (1:200; R&D systems, AF5105). After three washes with PBS (10 min each), samples were incubated for 1 h at room temperature in the dark with the following secondary antibodies from Proteintech: CoraLite 488‐conjugated goat anti‐rabbit IgG (SA00013‐2) or CoraLite 594‐conjugated goat anti‐mouse IgG (SA00013‐4). Images were acquired using a confocal microscope (Leica Microsystems, Buffalo Grove, IL, USA).

#### 4.9.2 Immunofluorescence Staining of Tissue Sections

The paraffin‐embedded tissue sections were deparaffinized and rehydrated, followed by antigen retrieval in 1 mM Tris‐EDTA buffer. To eliminate endogenous peroxidase interference, tissue sections were incubated with hydrogen peroxide, followed by the suppression of non‐specific binding using 5% bovine serum albumin. Sections were incubated with an anti‐Kla antibody at 4°C overnight. The next day, the sections were incubated with an HRP‐conjugated secondary antibody (SA00001‐2; Proteintech) for 30 min at 37°C, followed by incubation with Try‐488 fluorescence‐labeled reagent (Runnerbio, Shanghai, China). Subsequently, the sections were incubated with anti‐histone antibodies (H1, H2A, H2B, H3, and H4) and the corresponding secondary antibodies from Proteintech, followed by dual labeling using the Try‐Cy3 reagent. Finally, the sections were mounted with anti‐fade medium containing DAPI (P0131; Bristol‐Myers, Shnaghai, China) and imaged under a fluorescence microscope.

### 4.10 In Vitro Lactylation Assay

Recombinant HIS‐histone H3 (Ag10644, Proteintech, China) was incubated with 5 mM lactate (HY‐B2227, MCE, China) and recombinant DDX3x protein (Ag29972, Proteintech, China; 25–100 nM) in reaction buffer (pH 7.5, 25 mM KCl, 50 mM HEPES, 2 mM MgCl_2_, 4 mM ATP; 37°C, 3 h). The effects of Ode and the DDX3x mutants on the delactylase activity were evaluated under same reaction buffer. Following incubation, a 5 min thermal denaturation step at 95°C was subsequently executed on these samples prior to standard Western blot analysis.

### 4.11 Dot Bot Assay for Site‐Specific Histone Lactylation

Dot‐blot assays were performed using lactylated histone peptides to detect site‐specific histone lactylation. Synthetic peptides H3K18la (RKSTGG[Lys(lac)]APRK), H3K27la (ATKAAR[Lys(lac)]SAPATGG), and H3K9la (KQTAR[Lys(lac)] STGG) were purchased from Changsheng Biotechnology Co., Ltd. (China). Peptides were spotted onto a PVDF membrane, air‐dried, and blocked. Then, the membrane underwent overnight probing with primary antibody, a matching secondary antibody linked with HRP was subsequently applied.

### 4.12 GST Pulldown Assay

HIS‐DDX3x (CSB‐EP006621HUc7, CUSABIO, China) was incubated with GST‐tagged histone H3 (H00126961‐P01, Abnova, Taiwan) in GST buffer (pH 7.6, 50 mM HEPES,0.1% Nonidet P‐40, 50 mM NaCl, 5 mM EDTA, 10% glycerol) at 4°C for 12 h under gentle rotation. Precleared anti‐GST magnetic beads (FI8804; Fitgene, Guangzhou, China) were added to the mixture for an 4‐hour incubation. The beads were extensively washed with a high‐salt buffer (pH 8.0, 200 mM Tris‐Cl,0.1 mM EDTA, 500 mM NaCl, 0.4 mM PMSF, 0.1% Triton X‐100) to minimize nonspecific binding. Bound proteins were subsequently eluted and subjected to western blot analysis to detect protein interactions.

### 4.13 Cell Lysis and Co‐Immunoprecipitation (CO‐IP) Assay

HK‐2 cells were lysed on ice for 20 min using lysis buffer (50 mM Tris‐HCl, pH 7.4, 150 mM NaCl, 1% NP‐40, 1 mM EDTA, 10% glycerol, and 1 mM PMSF) supplemented with protease inhibitors. The lysates were centrifuged at 12 000 × *g* for 15 min at 4°C, and the supernatants were collected. Protein concentration was determined using the BCA assay. For each immunoprecipitation, 500–1000 µg of protein was incubated with a specific antibody or control IgG overnight at 4°C. Protein A/G beads were then added and incubation was continued for 1.5 h. To enable subsequent Western blot or mass spectrometry assessments, the captured target proteins underwent thermal elution with an appropriate loading buffer after the completion of four successive washes.

### 4.14 Chromatin Immunoprecipitation (ChIP) Assay

HK‐2 cells were cultured to logarithmic growth phase and cross‐linked with 1% formaldehyde containing protease inhibitors for 10 min at room temperature. The reaction was quenched by adding 125 mM glycine. After two washes with cold phosphate‐buffered saline (PBS), the cells were lysed, and chromatin was fragmented by sonication to 200–500 bp. The lysates were centrifuged and the supernatants were incubated with a target‐specific antibody overnight (PTM‐1419RM; PTM‐1406RM; PTM‐1428, PTM‐Bio) using a ChIP assay kit (17‐371RF, Sigma–Aldrich, USA). Pre‐washed protein A/G magnetic beads were then added, and incubation was continued for 2 h at 4°C. The immunocomplexes were sequentially washed with low‐salt, high‐salt, LiCl, and TE buffers according to the manufacturer's protocol. Following de‐crosslinking by proteinase K digestion, DNA was purified. The enrichment of the target DNA sequences was analyzed using PCR. All experiments were independently repeated at least thrice to ensure reproducibility. Table S1 comprehensively documents the specific oligonucleotide primer sequences utilized in this study.

### 4.15 Virtual Screening and Molecular Docking

The human DDX3x crystal structure (PDB: 8SSW) was retrieved from the Protein Data Bank. Hydrogen atoms were added and water, sulfate, chloride ions, ADP, and chain B were removed using the Protein Preparation Wizard. The structure was then energy‐minimized with the OPLS2005 force field to an RMSD of 0.30 Å. SiteMap identified five potential binding pockets; the top‐ranked site corresponded to the ADP‐binding pocket. As the goal was to discover agonists, the enzymatic active site was excluded, and subsequent screening focused on the allosteric pockets. Compounds from the HY‐L011P MCE 80K diversity library were prepared with LigPrep (hydrogen addition, energy minimization) to generate three‐dimensional conformers for virtual screening. The binding interface between DDX3x and histone H3 was predicted by protein–protein docking using the AlphaFold3 server.

### 4.16 Isothermal Titration Calorimetry (ITC) Assay

Isothermal titration calorimetry (ITC) assay was performed using a ITC calorimeter (MicroCal PEAQ, Malvern Panalytical) equipped with an automated sample‐handling system. 5 µM recombinant DDX3x protein (Ag29972, Proteintech, China) was loaded into the sample, while 50 µM Odetiglucan (HY‐147295, MCE) prepared in a buffer containing Tris‐HCl (50 mM) and NaCl (150 mM) was loaded into the syringe. For the titration program, a volume of 1 µl was introduced, followed by 19 individual delivery steps fixed at 2 µl. Injections were separated by a 300 s delay, while the stirring velocity was maintained at 500 rpm. Data processing and curve fitting were executed within the MicroCal‐supplied ORIGIN software.

### 4.17 RNA‐Sequencing Assay

Total RNA was isolated from mouse renal tissues using TRIzol reagent. The NEBNext Ultra RNA Library Prep Kit (NEB, #E7530) was utilized for the generation and assembly of RNA sequencing libraries. All experiments were performed in triplicate. Sequencing was performed on the DNBSEQ‐T7 platform using a paired‐end 150 bp (PE150) strategy. Following base calling using CASAVA, the raw sequence data were converted to the FASTQ format. Quality control and adapter trimming were performed using FastQC (v0.11.9) and Trimmomatic (v0.39). The STAR alignment engine (v2.7.10a) executed the mapping of cleaned reads against the mm10 mouse reference genome assembly, and the alignment files were processed using SAMtools (v1.16.1). Differential expression analysis was performed in R (v4.2.1) with the DESeq2 package (v1.20.0), applying a significance threshold of adjusted p < 0.05.

### 4.18 CUT&Tag

CUT&Tag assays were performed as described below. HK‐2 cells were harvested and incubated with Concanavalin A‐coated magnetic beads. Cells attached to the beads were sequentially incubated with primary antibody (anti‐H3K9/18/27la), secondary antibody, and hyperactive pA/G‐Tn5 transposase according to the manufacturer's protocol, followed by tagmentation. The DNA fragments were purified and amplified by PCR and the resulting products were cleaned. Libraries were constructed using the Hyperactive Universal CUT&T Ag Assay Kit from Illumina (Vazyme, Nanjing, China, #TD904) and sequenced on an Illumina NovaSeq 6000 platform.

Initial quality assessment of the newly generated CUT&Tag data workflow involved filtering the raw sequencing reads via FastQC software (v0.11.9). The Trimmomatic tool (v0.39) executed the automated elimination of both adapter contamination and substandard reads. The Bowtie2 alignment tool (v2.4.4) executed the mapping of filtered reads against the human reference genome. The resulting alignments were sorted using SAMtools (v1.9) and converted into the bedgraph format using the Genomecov function in Bedtools (v2.30.0). Peak visualization was performed using the IGV software (v2.14.1). The protocol established by the Steven Henikoff Laboratory guided the execution of subsequent analytical phase.

### 4.19 Data Analysis

GraphPad Prism (Version 9.0) facilitated all statistical computations, with experimental values expressed as the mean complemented by standard deviation (mean ± SD). For evaluating differences between two groups, an unpaired Student's t‐test was deployed; alternatively, for assessments involving multiple groups, a one‐way analysis of variance (ANOVA) was implemented, with subsequent group‐to‐group discrimination achieved through Tukey's post hoc test. Data significance was set at p < 0.05.

## Author Contributions

(**Hongyu Liang** and **Qiuyun Wang**) conceived/designed work; (Hongyu Liang, **Jihong Jiang** and **Huanxin Yin**) acquired/analyzed/interpreted data; (Hongyu Liang, Qiuyun Wang and **Minmin Zhu**) drafted manuscript; (**Yan Luo** and Minmin Zhu) reviewed manuscript; (**Hongjun Huang** and **Chunyan Liu**) revised the manuscript. Every contributing author has reviewed the manuscript and granted their consensus for its publication.

## Funding

The National Natural Science Foundation of China (No. 82272181 to Yan Luo; No. 82572464 to Qiuyun Wang) provided the financial support of this study.

## Conflicts of Interest

The authors declare no conflicts of interest.

## Supporting information




**Supporting File 1**: advs76979‐sup‐0001‐SuppMat.docx.

**Supporting File 2**: advs76979‐sup‐0002‐DataFile.xlsx.

## Data Availability

The data that support the findings of this study are available from the corresponding author upon reasonable request.
