# Supplementary material for: DDX3x Regulates NINJ1 Transcription via Histone Lactylation in Sepsis Associated‐Acute Kidney Injury
Source: Adv Sci (Weinh). 2026 Aug 17:e76979. Online ahead of print. doi: 10.1002/advs.76979 (PMC13479852; doi:10.1002/advs.76979)
Supplement: Supplementary file 1 [file ADVS-9999-e76979-s001.docx]

**Supplementary Figures and Figure legends**

**Title: DDX3x Regulates NINJ1 Transcription via Histone lactylation in Sepsis Associated-Acute Kidney Injury**

**Author: Hongyu Liang^1, #^, Jihong Jiang^2, #^, Huanxin Yin^3, #^, Chunyan Liu^1^, Hongjun Huang^1^, Yan Luo^1, *^, Minmin Zhu^4, *^, Qiuyun Wang^1, *^**

***Context:***

**Supplementary Fig.1----------------------------------------------------------------------------------------2**

**Supplementary Fig.1 legend-------------------------------------------------------------------------------3**

**Supplementary Fig.2----------------------------------------------------------------------------------------4**

**Supplementary Fig.2 legend-------------------------------------------------------------------------------5**

**Supplementary Fig.3----------------------------------------------------------------------------------------6**

**Supplementary Fig.3 legend-------------------------------------------------------------------------------7**

**Supplementary Fig.4----------------------------------------------------------------------------------------8**

**Supplementary Fig.4 legend-------------------------------------------------------------------------------9**

**Supplementary Fig.5---------------------------------------------------------------------------------------10**

**Supplementary Fig.5 legend------------------------------------------------------------------------------10**

**Supplementary Fig.6---------------------------------------------------------------------------------------11**

**Supplementary Fig.6 legend------------------------------------------------------------------------------12**

**Supplementary Fig.7---------------------------------------------------------------------------------------13**

**Supplementary Fig.7 legend------------------------------------------------------------------------------13**

**Supplementary Fig.8---------------------------------------------------------------------------------------14**

**Supplementary Fig.8 legend------------------------------------------------------------------------------15**

**Supplementary Fig.9---------------------------------------------------------------------------------------16**

**Supplementary Fig.9 legend------------------------------------------------------------------------------17**

**Supplementary Fig.10 -------------------------------------------------------------------------------------18**

**Supplementary Fig.10 legend ----------------------------------------------------------------------------19**

**Supplementary Fig.11 -------------------------------------------------------------------------------------20**

**Supplementary Fig.11 legend ----------------------------------------------------------------------------21**

**Supplementary Fig.12 -------------------------------------------------------------------------------------22**

**Supplementary Fig.12 legend ----------------------------------------------------------------------------23**

**Supplementary Fig.13 -------------------------------------------------------------------------------------24**

**Supplementary Fig.13 legend ----------------------------------------------------------------------------25**

**Supplementary Fig.14 -------------------------------------------------------------------------------------26**

**Supplementary Fig.14 legend ----------------------------------------------------------------------------27**

**Supplementary Fig.15 -------------------------------------------------------------------------------------28**

**Supplementary Fig.15 legend ----------------------------------------------------------------------------29**

**Supplementary Fig.16 -------------------------------------------------------------------------------------30**

**Supplementary Fig.16 legend ----------------------------------------------------------------------------31**

**Supplementary Fig.17 -------------------------------------------------------------------------------------32**

**Supplementary Fig.17 legend ----------------------------------------------------------------------------33**

**Supplementary Fig.1**

**Supplementary Fig. 1 The levels of lactate, lysine lactylation (Kla), H3K9la, H3K18la and H3K27la were increased in LPS treated HK-2 cells**

A: Analysis of glycolysis-related genes (HK-1, HIF-1A, LDHA, PDHA, PKM) in the GSE95233 database.

B: Western blotting revealed that Pan-Kla reached its peak expression following 12 hours of LPS treatment at a concentration of 10 μg/mL in HK-2 cells. (n=4)

C: Western blotting revealed that H3K9la, H3K18la and H3K27la reached its peak expression following 12 hours of LPS treatment at a concentration of 10 μg/mL in HK-2 cells. (n=4)

D: Lactate levels were determined in HK-2 cells. Compared to Con group, LPS treated HK-2 cells exhibited elevated lactate levels. (n=4)

E: Immunoprecipitation of H1, H2A, H2B, H3, and H4 in HK-2 cells treated with Con and LPS respectively revealed a significant increase in the lactylation level of H3 following LPS treatment. (n=4)

F: Western blotting revealed that compared to Con group, the levels of Pan-Kla, H3K9la, H3K18la and H3K27la were significantly increased in LPS treated HK-2 cells. (n=4)

G: LDH release was employed to assess the extent of cell death. Compared to the Con group, LPS treated HK-2 cells exhibited higher LDH release. (n=4)

H: Representative PI staining images. Compared to the Con group, LPS group exhibited more dead cells in HK-2 cells (red indicates dead cells, green indicates viable cells). (Scale Bar:100μm, n=4) *P<0.05, **P<0.01, ***P<0.001, and ****P<0.0001.

**Supplementary Fig.2**

**Supplementary Fig.2 Inhibiting lactate production using DCA can decrease H3K9/18/27la and cell death**

A: Representative immunohistochemical (IHC) staining for Pan-Kla, H3K9la, H3K18la and H3K27la was performed on renal tissue sections from sham, DCA, CLP, CLP+DCA mice. Compared with CLP mice, the CLP+DCA group exhibited lower level of Pan-Kla, H3K9la, H3K18la and H3K27la. And representative microscopic images display haematoxylin and eosin (HE) and Masson's trichrome staining of renal tissue from the sham, DCA, CLP, CLP+DCA mice. Compared to the CLP group, CLP+DCA mice exhibited milder tubular swelling and damage. (Scale Bar:50μm, n=8).

B: Lactate levels were determined in renal tissue. Compared to CLP group mice, CLP+DCA mice exhibited decreased lactate levels. (n=8)

C: Western blotting revealed that compared to CLP mice, the levels of Pan-Kla, H3K27la, H3K18la and H3K9la were significantly reduced in CLP+DCA mice. (n=8)

D: Levels of the inflammatory mediators IL-1β and TNF-α in the Sham, DCA, CLP and CLP+DCA groups. (n=8)

E: Levels of renal injury markers BUN and Scr in the in the Sham, DCA, CLP and CLP+DCA groups. (n=8)

F: Western blotting revealed that compared to LPS grouop, the levels of Pan-Kla, H3K27la, H3K18la and H3K9la were significantly reduced in LPS+DCA group. (n=4)

G: Lactate levels were determined in HK-2 cells. Compared to LPS group, LPS+DCA mice exhibited decreased lactate levels. (n=4)

H: LDH release was employed to assess the extent of cell death. Compared to the LPS group, the LPS+DCA group exhibited lower LDH release in HK-2 cells. (n=4)

I: Representative PI staining images. Compared to the LPS group, the LPS+DCA group exhibited reduced cell death (red indicates dead cells, green indicates viable cells). (Scale Bar:100μm, n=4) *P<0.05, **P<0.01, ***P<0.001, and ****P<0.0001

**Supplementary Fig.3**

**Supplementary Fig.3 Supplementation with sodium lactate increases H3K9la, H3K18la, H3K27la and cell death**

A: Representative immunohistochemical (IHC) staining for Pan-Kla, H3K9la, H3K18la and H3K27la was performed on renal tissue sections from sham, Nala, CLP, CLP+Nala mice. Compared with CLP mice, the CLP+Nala group exhibited higher level of Pan-Kla, H3K9la, H3K18la and H3K27la. And representative microscopic images display haematoxylin and eosin (HE) and Masson’s trichrome staining of renal tissue from the sham, Nala, CLP, CLP+Nala mice. Compared to the CLP group, CLP+Nala mice exhibited more severe tubular swelling and damage. (Scale Bar:50μm, n=8)

B: Levels of the inflammatory mediators IL-1β and TNF-α in the Sham, Nala, CLP and CLP+Nala groups. (n=8)

C: Levels of renal injury markers BUN and Scr in the in the Sham, Nala, CLP and CLP+Nala groups. (n=8)

D: Western blotting revealed that compared to CLP mice, the levels of Pan-Kla, H3K27la, H3K18la and H3K9la were significantly reduced in CLP+Nala mice. (n=8)

E: Western blotting revealed that compared to LPS group, the levels of Pan-Kla, H3K27la, H3K18la and H3K9la were increased in LPS+Nala group. (n=4)

F: LDH release was employed to assess the extent of cell death. Compared to the LPS group, the LPS+Nala group exhibited higher LDH release in HK-2 cells. (n=4)

G: Representative PI staining images. Compared to the LPS group, the LPS+Nala group exhibited more dead cells (red indicates dead cells, green indicates viable cells). (Scale Bar:100μm, n=4) *P<0.05, **P<0.01, ***P<0.001, and ****P<0.0001.

**Supplementary Fig.4**

**Supplementary Fig.4 H3K9R, H3K18R and H3K27R can protect against LPS-induced cell death**

A: LDH release was employed to assess the extent of cell death. Compared to the WT-H3 group, the K9R-H3 group exhibited lower LDH release in LPS treated HK-2 cells. (n=4)

B: Representative PI staining images. Compared to the WT-H3 group, the K9R-H3 group exhibited reduced cell death (red indicates dead cells, green indicates viable cells). (Scale Bar:100μm, n=4)

C: LDH release was employed to assess the extent of cell death. Compared to the WT-H3 group, the K18R-H3 group exhibited lower LDH release in LPS treated HK-2 cells. (n=4)

D: Representative PI staining images. Compared to the WT-H3 group, the K18R-H3 group exhibited reduced cell death (red indicates dead cells, green indicates viable cells). (Scale Bar:100μm, n=4)

E: LDH release was employed to assess the extent of cell death. Compared to the WT-H3 group, the K27R-H3 group exhibited lower LDH release in LPS treated HK-2 cells. (n=4)

F: Representative PI staining images. Compared to the WT-H3 group, the K27R-H3 group exhibited reduced cell death (red indicates dead cells, green indicates viable cells). (Scale Bar:100μm, n=4) *P<0.05, **P<0.01, ***P<0.001, and ****P<0.0001.

**Supplementary Fig.5**

**Supplementary Fig.5 Significant increases in PANoptosis-associated proteins were observed in CLP mice**

A: KEGG analysis revealed that pathways associated with cellular PANoptosis (NOD-like receptor signaling pathway, TNF signaling pathway, Cytosolic DNA-sensing pathway, Apoptosis, Necroptosis) were significantly enriched.

B: Western blotting revealed that compared to sham group, the levels of PANoptosis related protein (C-CASP1, N-GSDMD, C-CASP3, C-CASP8, P-MLKL, P-RIPK1) were significantly increased in CLP mice. (n=8)

C: Western blotting revealed that compared to Con group, the levels of PANoptosis related protein (C-CASP1, N-GSDMD, C-CASP3, C-CASP8, P-MLKL, P-RIPK1) were significantly increased in LPS treated HK-2 cells. (n=8) *P<0.05, **P<0.01, ***P<0.001, and ****P<0.0001.

**Supplementary Fig.6**

**Supplementary Fig.6 Inhibiting lactate production can significantly reduce the elevation of PANoptosis caused by sepsis.**

A: Western blotting revealed that compared to CLP group, the levels of PANoptosis related protein (C-CASP1, N-GSDMD, C-CASP3, C-CASP8, P-MLKL, P-RIPK1) were significantly decreased in CLP+Oxa group. (n=8)

B: Western blotting revealed that compared to LPS group, the levels of PANoptosis related protein (C-CASP1, N-GSDMD, C-CASP3, C-CASP8, P-MLKL, P-RIPK1) were significantly decreased in LPS+Oxa group. (n=4)

C: Western blotting revealed that compared to CLP group, the levels of PANoptosis related protein (C-CASP1, N-GSDMD, C-CASP3, C-CASP8, P-MLKL, P-RIPK1) were significantly decreased in CLP+DCA group. (n=8)

D: Western blotting revealed that compared to LPS group, the levels of PANoptosis related protein (C-CASP1, N-GSDMD, C-CASP3, C-CASP8, P-MLKL, P-RIPK1) were significantly decreased in LPS+DCA group. (n=4) *P<0.05, **P<0.01, ***P<0.001, and ****P<0.0001.

**Supplementary Fig.7**

**Supplementary Fig.7 Supplementation with sodium lactate increases PANoptosis**

A: Western blotting revealed that compared to CLP group, the levels of PANoptosis related protein (C-CASP1, N-GSDMD, C-CASP3, C-CASP8, P-MLKL, P-RIPK1) were significantly increased in CLP+Nala group. (n=8)

B: Western blotting revealed that compared to WT-H3 group, the levels of PANoptosis related protein (C-CASP1, N-GSDMD, C-CASP3, C-CASP8, P-MLKL, P-RIPK1) were significantly increased in LPS+Nala group. (n=4) *P<0.05, **P<0.01, ***P<0.001, and ****P<0.0001.

**Supplementary Fig.8**

**Supplementary Fig.8 H3K9la, H3K18la and H3K27la regulate NINJ1 gene transcription**

A: Chromatin immunoprecipitation-PCR (CHIP-PCR) revealed enrichment of H3K9la, H3K18la and H3K27la in the promoter region of the NINJ1 gene. (n=3)

B: Volcano plot of NINJ1 gene expression. Red indicates upregulated genes, blue indicates downregulated genes.

C: Cell clustering map derived from UMAP-based single-cell annotation of the GSE199321 dataset.

D: Bubble plot of characteristic gene expression across different cell subpopulations from GSE199321.

E: Differential expression of NINJ1 across distinct cell subpopulations. Cells highlighted in red represent renal tubular epithelial cells.

F: Western blotting revealed that compared to WT-H3 group, the K9R-H3 group exhibited lower levels of NINJ1 protein in LPS treated HK-2 cells. (n=4)

G: qPCR assay indicated that compared to WT-H3 group, K9R-H3 group exhibited lower levels of NINJ1 mRNA levels in LPS treated HK-2 cells. (n=4)

H: Western blotting revealed that compared to WT-H3 group, the K18R-H3 group exhibited lower levels of NINJ1 protein in LPS treated HK-2 cells. (n=4)

I: qPCR assay indicated that compared to WT-H3 group, K18R-H3 group exhibited lower levels of NINJ1 mRNA levels in LPS treated HK-2 cells. (n=4)

J: Western blotting revealed that compared to WT-H3 group, the K27R-H3 group exhibited lower levels of NINJ1 protein in LPS treated HK-2 cells. (n=4)

K: qPCR assay indicated that compared to WT-H3 group, K27R-H3 group exhibited lower levels of NINJ1 mRNA levels in LPS treated HK-2 cells. (n=4)

L: Co-expression network analysis of the NINJ1 gene.

M: The first ChIP-qPCR was performed using antibodies against H3K9la, H3K18la, and H3K27la in cells from the Con and LPS groups. (n=4)

N: Sequential ChIP-qPCR: enrichment with anti-H3K9la, anti-H3K18la, or anti-H3K27la, followed by a second enrichment with anti-Zhx2. (n=4)*P<0.05, **P<0.01, ***P<0.001, and ****P<0.0001.

**Supplementary Fig.9**

**Supplementary Fig.9 Knocking down NINJ1 significantly ameliorates LPS-induced cell death**

A: Representative immunofluorescence images from AAV9-ZsGreen, AAV-shNinj1, CLP+AAV9-ZsGreen, CLP+ AAV-shNinj1 groups, reveal AAV9-mediated tubular-specific knockdown of NINJ1. (Scale Bar:50μm, n=8)

B: qPCR assay indicated that compared to CLP+AAV9-ZsGreen group, CLP+ AAV-shNinj1 group exhibited lower levels of NINJ1 mRNA levels. (n=8)

C: Western blotting revealed that compared to LPS group, the LPS+si^#1^-Ninj1 and LPS+si^#2^-Ninj1 group exhibited lower levels of NINJ1 protein in HK-2 cells. (n=4)

D: qPCR assay indicated that compared to LPS group, LPS+si^#1^-Ninj1 and LPS+si^#2^-Ninj1 group exhibited lower levels of NINJ1 mRNA levels. (n=4)

E: LDH release was employed to assess the extent of cell death. Compared to the LPS group, the LPS+si^#1^-Ninj1 and LPS+si^#2^-Ninj1 group exhibited lower LDH release in HK-2 cells. (n=4)

F: Western blotting revealed that compared to LPS group, the levels of PANoptosis related protein (C-CASP1, N-GSDMD, C-CASP3, C-CASP8, P-MLKL, P-RIPK1) were significantly reduced in LPS+si^#1^-Ninj1 and LPS+si^#2^-Ninj1 group. (n=4) *P<0.05, **P<0.01, ***P<0.001, and ****P<0.0001.

**Supplementary Fig.10**

**Supplementary Fig.10 DDX3x expression is reduced during SA-AKI**

A: GO analysis revealed that RNA binding protein was significantly enriched.

B: Representative immunohistochemical (IHC) staining for DDX3x was performed on renal tissue sections from sham and CLP mice. Compared to sham mice, the CLP group exhibited lower level of DDX3x protein (Scale Bar:50μm, n=8).

C: Western blotting revealed that compared to sham group, the levels of DDX3x was significantly reduced in CLP group. (n=8)

D: qPCR assay indicated that compared to sham group, CLP group exhibited lower levels of DDX3x mRNA levels. (n=8)

E: Western blotting revealed that compared to Con group, the levels of DDX3x was significantly reduced in LPS treated HK-2 cells. (n=4)

F: qPCR assay indicated that compared to Con group, LPS group exhibited lower levels of DDX3x mRNA levels. (n=4)

G: Differential expression of DDX3x across distinct cell subpopulations. Cells highlighted in red represent renal tubular epithelial cells. *P<0.05, **P<0.01, ***P<0.001, and ****P<0.0001.

**Supplementary Fig.11**

**Supplementary Fig.11 DDX3x N537R/K418Q mutation significantly reduces its delactyltransferase activity**

A: Schematic diagram of the DDX3x N537R/K418Q mutation.

B: Immunoprecipitation assay revealed that the mutation significantly reduced the binding capacity of DDX3x to H3K9la, H3K18la, and H3K27la. (n=4)

C: Isothermal titration calorimetry (ITC) analysis of the interaction between purified wild-type (WT) DDX3x and purified histone H3.

D: ITC analysis of the interaction between purified MUT-DDX3x and purified histone H3.

E: An *in vitro* lactylation assay showed that compared to WT-DDX3x, MUT-DDX3x exhibited lower delactyltransferase activity to H3K9la, H3K18la and H3K27la. (n=4)

F: Western blotting revealed that compared to WT-DDX3x group, the levels of H3K9la, H3K18la and H3K27la was significantly reduced in LPS treated HK-2 cells. (n=4)

G: Following the separation of nucleus (NC) proteins from cytoplasmic (CY) proteins, compared to the Con group, LPS group exhibited reduced DDX3x nuclear localization. (n=4)

H: Western blotting analysis of H3K9la, H3K18la, and H3K27la levels in HK-2 cells after individual transfection with Vector^Flag^, DDX3x^Flag^, HDAC1–3^Flag^, or Sirt1–3^Flag^. (n=4) *P<0.05, **P<0.01, ***P<0.001, and ****P<0.0001.

**Supplementary Fig.12**

**Supplementary Fig.12 Overexpression of DDX3x in LPS-induced cells significantly reduces NINJ1 expression and PANoptosis**

A: Western blotting revealed that compared to LPS group, the levels of NINJ1 was significantly reduced, and DDX3x was significantly increased in LPS+DDX3x-OE group. (n=4)

B: qPCR assay indicated that compared to LPS group, LPS+DDX3x-OE group exhibited higher levels of DDX3x mRNA levels. (n=4)

C: qPCR assay indicated that compared to LPS group, LPS+DDX3x-OE group exhibited lower levels of NINJ1 mRNA levels. (n=4)

D: LDH release was employed to assess the extent of cell death. Compared to the LPS group, the LPS+DDX3x-OE group exhibited lower LDH release in HK-2 cells. (n=4)

E: Representative PI staining images. Compared to the LPS group, the LPS+DDX3x-OE group exhibited reduced cell death (red indicates dead cells, green indicates viable cells). (Scale Bar:100μm, n=4).

F: Western blotting revealed that compared to LPS group, the levels of PANoptosis related protein (C-CASP1, N-GSDMD, C-CASP3, C-CASP8, P-MLKL, P-RIPK1) were significantly reduced in LPS+DDX3x-OE group. (n=4) *P<0.05, **P<0.01, ***P<0.001, and ****P<0.0001.

**Supplementary Fig.13**

**Supplementary Fig.13 Knockdown of DDX3x leads to increased level of NINJ1 and PANoptosis**

A: Western blotting revealed that compared to Con group, the levels of DDX3x was significantly reduced, and NINJ1 was significantly increased in si^#1^-DDX3x and si#2-DDX3x group. (n=4)

B: qPCR assay indicated that compared to Con group, si^#1^-DDX3x and si^#2^-DDX3x group exhibited lower levels of DDX3x mRNA levels. (n=4)

C: qPCR assay indicated that compared to Con group, si^#1^-DDX3x and si^#2^-DDX3x group exhibited higher levels of NINJ1 mRNA levels. (n=4)

D: LDH release was employed to assess the extent of cell death. Compared to the Con group, si^#1^-DDX3x and si^#2^-DDX3x group exhibited higher LDH release in HK-2 cells. (n=4)

E: Representative PI staining images. Compared to the Con group, si^#1^-DDX3x and si^#2^-DDX3x group exhibited more dead cells (red indicates dead cells, green indicates viable cells). (Scale Bar:100μm, n=4).

F: Western blotting revealed that compared to Con group, the levels of PANoptosis related protein (C-CASP1, N-GSDMD, C-CASP3, C-CASP8, P-MLKL, P-RIPK1) were significantly increased in si#1-DDX3x and si#2-DDX3x group. (n=4) *P<0.05, **P<0.01, ***P<0.001, and ****P<0.0001.

**Supplementary Fig.14**

**Supplementary Fig.14 In HK-2 cells, dual knockdown of NINJ1 and DDX3x significantly alleviates the increase in NINJ1 and PANoptosis induced by DDX3x knockdown**

A: Western blotting revealed that compared to si-DDX3x group, the levels of NINJ1 was significantly reduced in si-DDX3x+si-NINJ1 group. (n=4)

B: qPCR assay indicated that compared to si-DDX3x group, si-DDX3x+si-NINJ1 group exhibited no significant change of DDX3x mRNA levels. (n=4)

C: qPCR assay indicated that compared to si-DDX3x group, si-DDX3x+si-NINJ1 group exhibited lower level of NINJ1 mRNA levels. (n=4)

D: LDH release was employed to assess the extent of cell death. Compared to the si-DDX3x group, si-DDX3x+si-NINJ1 group exhibited lower LDH release in HK-2 cells. (n=4)

E: Representative PI staining images. Compared to the si-DDX3x group, si-DDX3x+si-NINJ1 group exhibited lower cell death (red indicates dead cells, green indicates viable cells). (Scale Bar:100μm, n=4).

F: Western blotting revealed that compared to si-DDX3x group, the levels of PANoptosis related protein (C-CASP1, N-GSDMD, C-CASP3, C-CASP8, P-MLKL, P-RIPK1) were significantly decreased in si-DDX3x+si-NINJ1 group. (n=4) *P<0.05, **P<0.01, ***P<0.001, and ****P<0.0001.

**Supplementary Fig.15**

**Supplementary Fig.15 The DDX3xN537R/K418Q mutation markedly diminishes the protective effect of WT-DDX3x against LPS-induced PANoptosis**

A: LDH release was employed to assess the extent of cell death. Compared to the WT-DDX3x group, MUT-DDX3x group exhibited higher LDH release in LPS treated HK-2 cells. (n=4)

B: Representative PI staining images. Compared to WT-DDX3x group, MUT-DDX3x group exhibited more dead cells in LPS treated HK-2 cells (red indicates dead cells, green indicates viable cells). (Scale Bar:100μm, n=4).

C: Western blotting revealed that compared to WT-DDX3x group, the levels of PANoptosis related protein (C-CASP1, N-GSDMD, C-CASP3, C-CASP8, P-MLKL, P-RIPK1) were significantly increased in MUT-DDX3x group. (n=4) *P<0.05, **P<0.01, ***P<0.001, and ****P<0.0001.

**Supplementary Fig.16**

**Supplementary Fig.16 Ode reduced LPS-induced NINJ1 expression and PANoptosis in cells**

A: Molecular dynamics simulation (0–100 ns) of the binding conformation between Ode and DDX3x.

B: Molecular dynamics simulation showing changes in RMSD and Rg values of the DDX3x–Ode complex over 0–100 ns.

C: ITC analysis of Ode binding to DDX3x.

D: *In vitro* lactylation assays indicate that Ode can activate the delactyltransferase activity of DDX3x in a dose-dependent manner. (n=4)

E: Western blotting revealed that Ode can reduced H3K9la, H3K18la, H3K27la and NINJ1 level in LPS treated HK-2 cells in a dose-dependent manner. (n=4)

F: qPCR assay indicated that compared to LPS group, LPS+Ode group exhibited lower level of NINJ1 mRNA levels. (n=4)

G: qPCR assay indicated that Ode treatment was not change DDX3x mRNA level. (n=4)

H: Western blotting revealed that Ode can reduced NINJ1 level without DDX3x levels change in LPS treated HK-2 cells. (n=4)

I: LDH release was employed to assess the extent of cell death. Compared to the LPS group, the LPS+Ode group exhibited lower LDH release in HK-2 cells. (n=4)

J: Western blotting revealed that compared to LPS group, the levels of PANoptosis related protein (C-CASP1, N-GSDMD, C-CASP3, C-CASP8, P-MLKL, P-RIPK1) were significantly decreased in LPS+Ode group. (n=4)

K: Seven-day survival of Sham, Ode, CLP, and CLP + Ode mice. (n=8)

L: Western blot showing H3K9la, H3K18la, H3K27la, and NINJ1 levels in Con, Ode, LPS, and LPS + Ode groups. (n=4) *P<0.05, **P<0.01, ***P<0.001, and ****P<0.0001.

**Supplementary Fig.17**

**Supplementary Fig.17 Ode also alleviates lung injury in CLP mice**

A: Western blotting revealed that compared to CLP mice, the levels of H3K27la, H3K18la, H3K9la were significantly reduced without change DDX3x level in CLP+Ode mice lung. (n=8)

B: qPCR assay indicated decreased DDX3x mRNA levels in CLP mice lung compared to sham mice lung. (n=8)

C: Representative immunohistochemical (IHC) staining for Pan-Kla, H3K9la, H3K18la and H3K27la was performed on lung tissue sections from sham, sham+Ode, CLP, CLP+Ode mice. Compared with CLP, CLP+Ode group exhibited lower level of Pan-Kla, H3K9la, H3K18la and H3K27la (Scale Bar:50μm, n=8).

D: Representative microscopic images display haematoxylin and eosin (HE) and immunohistochemical staining for NINJ1 of lung tissue from the sham, sham+Ode, CLP, CLP+Ode mice. Compared to the CLP mice, CLP+Ode exhibited milder alveolar wall injury, and lower NINJ1 protein level (Scale Bar:50μm, n=8).

E: Western blotting revealed that compared to CLP mice, the levels of NINJ1 was significantly reduced in CLP+Ode mice lung. (n=8)

F: qPCR assay indicated decreased NINJ1 mRNA levels in CLP+Ode mice lung compared to CLP mice lung. (n=8)

G: Wet to Dry ratio is employed to assess the severity of pulmonary injury. Compared with the CLP group, Wet to Dry ratio improved in the CLP+Ode group. (n=8)

H: Lung injury score is employed to assess the severity of pulmonary injury. Compared with the CLP group, lung injury score decreased in the CLP+Ode group. (n=8)

I: Western blotting revealed that compared to CLP group, the levels of PANoptosis related protein (C-CASP1, N-GSDMD, C-CASP3, C-CASP8, P-MLKL, P-RIPK1) were significantly reduced in CLP+Ode mice. (n=8)

J: Relative levels of cTnT in the Sham, Ode, CLP and CLP+Ode groups. (n=8)

K: Relative levels of BNP in the Sham, Ode, CLP and CLP+Ode groups. (n=8)

L: Relative levels of ALT in the Sham, Ode, CLP and CLP+Ode groups. (n=8) *P<0.05, **P<0.01, ***P<0.001, and ****P<0.0001.
